# Competition between maturation and degradation drives human snRNA 3′ end quality control

**DOI:** 10.1101/gad.336891.120

**Published:** 2020-07-01

**Authors:** Rea M. Lardelli, Jens Lykke-Andersen

**Affiliations:** Division of Biological Sciences, University of California San Diego, La Jolla, California 92093, USA

**Keywords:** TOE1, Caf1z, snRNA biogenesis, snRNA quality control, adenylation, deadenylation, pontocerebellar hypoplasia

## Abstract

Polymerases and exonucleases act on 3′ ends of nascent RNAs to promote their maturation or degradation but how the balance between these activities is controlled to dictate the fates of cellular RNAs remains poorly understood. Here, we identify a central role for the human DEDD deadenylase TOE1 in distinguishing the fates of small nuclear (sn)RNAs of the spliceosome from unstable genome-encoded snRNA variants. We found that TOE1 promotes maturation of all regular RNA polymerase II transcribed snRNAs of the major and minor spliceosomes by removing posttranscriptional oligo(A) tails, trimming 3′ ends, and preventing nuclear exosome targeting. In contrast, TOE1 promotes little to no maturation of tested U1 variant snRNAs, which are instead targeted by the nuclear exosome. These observations suggest that TOE1 is positioned at the center of a 3′ end quality control pathway that selectively promotes maturation and stability of regular snRNAs while leaving snRNA variants unprocessed and exposed to degradation in what could be a widespread mechanism of RNA quality control given the large number of noncoding RNAs processed by DEDD deadenylases.

## Abstract (precis)

Here, Lardelli and Lykke-Andersen investigated the control of cellular RNA fates by studying the balance between polymerases and exonucleases, which act on 3′ ends of nascent RNAs to promote their maturation or degradation. They identified a central role for the human DEDD deadenylase TOE1 in distinguishing the fates of small nuclear (sn)RNAs of the spliceosome from unstable genome-encoded snRNA variants, and their findings provide new mechanistic insights into RNA quality control.

A large number of polymerases and exonucleases act on RNA 3′ ends to control the destiny of newly transcribed RNAs, promoting their maturation or degradation. A variety of polymerases have been implicated in eukaryotic RNA maturation processes such as the polyadenylation of mRNAs (Darnell et al. 1971; Edmonds et al. 1971; Lee et al. 1971), tRNA CCA addition (Sprinzl and Cramer 1979; Deutscher 1982), and uridylation of U6 small nuclear (sn)RNA (Reddy et al. 1987). Other polymerases promote RNA degradation including polymerases of the TRAMP complex that link RNA oligoadenylation to 3′-to-5′ degradation by the nuclear exosome (LaCava et al. 2005; Vaňáčová et al. 2005; Wyers et al. 2005) and terminal uridine transferases (TUTases) that add oligouridine tails to target RNAs for degradation (Rissland and Norbury 2009). In a similar manner, an assortment of 3′-to-5′ exonucleases promote maturation or degradation depending on the specific enzyme, RNA, and context (Ibrahim et al. 2008; Zinder and Lima 2017). The rules that dictate whether a newly synthesized RNA is destined for maturation or degradation by these competing activities remain poorly understood.

Recent studies have identified 3′-to-5′ exonucleases that belong to the DEDD family of deadenylases as critical for the maturation of a variety of small noncoding RNAs. The initial discovery was a role for the deadenylase PARN in the processing of small nucleolar (sno)RNAs (Berndt et al. 2012). PARN was subsequently found to also process other RNAs including telomerase RNA (Moon et al. 2015; Tseng et al. 2015; Shukla et al. 2016; Son et al. 2018; Roake et al. 2019), small Cajal Body associated (sca)RNAs (Son et al. 2018), Y-RNAs (Shukla and Parker 2017), and miRNAs (Yoda et al. 2013; Shukla and Parker 2017). More recently, the PARN homolog PNLDC1 was found to process piwi (pi)RNAs (Ding et al. 2017; Zhang et al. 2017; Nishimura et al. 2018), and a more distant homolog of PARN, TOE1, was shown in human cells to process small nuclear (sn)RNAs (Lardelli et al. 2017; Son et al. 2018), and along with PARN, to process snoRNAs, scaRNAs (Son et al. 2018), and telomerase RNA (Son et al. 2018; Deng et al. 2019). RNA 3′ end adenylation is thought to play a role in these maturation processes based on the accumulation of extended precursor RNAs that are often oligoadenylated upon depletion or mutation of catalytic residues of these enzymes (Berndt et al. 2012; Lardelli et al. 2017; Shukla and Parker 2017; Son et al. 2018). The importance of the DEDD family deadenylases is underscored by genetic mutations in *PARN* and *TOE1* genes leading to specific subtypes of human disorders dyskeratosis congenita and pontocerebellar hypoplasia (PCH), respectively (Dhanraj et al. 2015; Stuart et al. 2015; Tummala et al. 2015; Lardelli et al. 2017).

One class of small RNAs processed by a DEDD family deadenylase is RNA polymerase II transcribed snRNAs, which undergo a complex maturation pathway before forming the catalytic core of the spliceosome. These RNAs are cotranscriptionally ^m7^G-capped at the 5′ end by capping and methylation enzymes (Salditt-Georgieff et al. 1980) and cleaved at the 3′ end by the Integrator complex (Baillat et al. 2005), which leaves a short genome-encoded 3′ tail. They are then exported to the cytoplasm by the export adapter PHAX (Ohno et al. 2000), where they undergo assembly with the Sm complex regulated by SMN and Gemin proteins in conjunction with protein arginine methyl transferases (PRMTs) (Fischer et al. 1997; Liu et al. 1997; Buhler et al. 1999; Friesen et al. 2001; Meister et al. 2001a,b; Massenet et al. 2002; Pellizzoni et al. 2002). Following Sm complex assembly, the 5′ cap is trimethylated also in the cytoplasm (Mattaj 1986), which serves as a signal for nuclear import by Snurportin (SNUPN) and Importin β (Palacios et al. 1997; Huber et al. 1998). snRNAs subsequently undergo scaRNA-directed 2′-O-methylation and pseudouridylation (Reddy and Busch 1988) and snRNA-specific protein (snRNP) assembly to form complexes active in pre-mRNA splicing.

While much has been learned about snRNA biogenesis, the mechanism and importance of the processing of snRNA 3′ ends that occurs after Integrator cleavage remain poorly understood despite having been first observed >30 yr ago (Eliceiri and Sayavedra 1976; Madore et al. 1984). Early evidence from *Xenopus laevis* oocytes injected with precursor snRNAs demonstrated processing of 3′-terminal nucleotides upon nuclear import (Yang et al. 1992), yet the responsible nuclease remained unidentified. In the budding yeast *Saccharomyces cerevisiae*, snRNA 3′ end trimming is carried out by the exosome (Allmang et al. 1999; Seipelt et al. 1999), and a recent study found that budding yeast precursor snRNAs deficient in 3′ end maturation can assemble into spliceosomes but cause widespread splicing defects (Becker et al. 2019). We recently identified the DEDD family deadenylase TOE1 as an enzyme critical for snRNA 3′ end trimming in human cells (Lardelli et al. 2017). Depletion of TOE1 had been previously observed to result in a pre-mRNA splicing defect (Fong et al. 2013) but how TOE1-mediated snRNA 3′ end processing is integrated with snRNP biogenesis has remained unknown.

In addition to the regular snRNAs of the spliceosome that accumulate at high levels and participate in pre-mRNA splicing, hundreds of snRNA variants are encoded in the mammalian genome (Denison et al. 1981; Chen et al. 2005; Marz et al. 2008; O'Reilly et al. 2013) some of which have high sequence conservation in long flanking regions and are presumed to have arisen from gene duplication (Denison and Weiner 1982) while others lacking flanking homology are presumed to have arisen through RNA-mediated mechanisms. Some of these variants are up-regulated in specific developmental stages and tissues, where they may have specialized functions (Lund et al. 1985; Lo and Mount 1990; Jia et al. 2012; O'Reilly et al. 2013), but the transcriptional status and fates of the vast majority of snRNA variants are unknown. In the case of human U1 snRNA variants, some have identical or near-identical sequence to regular U1 snRNA produced from *U1.1-4* snRNA genes and likely participate in normal pre-mRNA splicing. Others are highly transcribed as evidenced by chromatin immunoprecipitation assays for active transcription machinery, but accumulate at low levels suggesting they are rapidly degraded (O'Reilly et al. 2013). While quality control pathways that target snRNAs with introduced mutations or long 3′ end extended snRNAs resulting from defects in transcription termination have been described (Shukla and Parker 2014; Hrossova et al. 2015; Łabno et al. 2016; Pirouz et al. 2016; Ustianenko et al. 2016), how endogenous unstable snRNA variants are discriminated from regular snRNAs and maintained at low cellular levels remains unknown.

Here, we investigated the role of the DEDD family deadenylase TOE1 in the biogenesis of regular and variant snRNAs. By monitoring effects of TOE1 depletion on nascent snRNA accumulation, biogenesis factor association, and 3′ end processing, we found that TOE1 processes major and minor class snRNAs in at least two stages of biogenesis, before or during their association with the nuclear export factor PHAX and again, during or after association with nuclear import machinery. TOE1 depletion causes accumulation of extended snRNA intermediates that are heavily adenylated, accumulate at aberrantly high levels with the nuclear export factor PHAX, and increase in levels upon depletion of nuclear exosome factors suggesting that TOE1 promotes snRNA biogenesis in competition with degradation in the nucleus. In sharp contrast, TOE1 promotes little to no processing of tested U1 variant snRNAs, which are instead rapidly degraded, at least in part by the nuclear exosome. These findings suggest that TOE1 is positioned at the center of an snRNA quality control pathway in which TOE1 specificity drives the equilibrium between oligoadenylating and exonucleolytic activities, promoting maturation of regular snRNAs while exposing unstable snRNA variants to degradation.

## Results

### TOE1 processes major and minor class snRNAs

**Figure 1.**
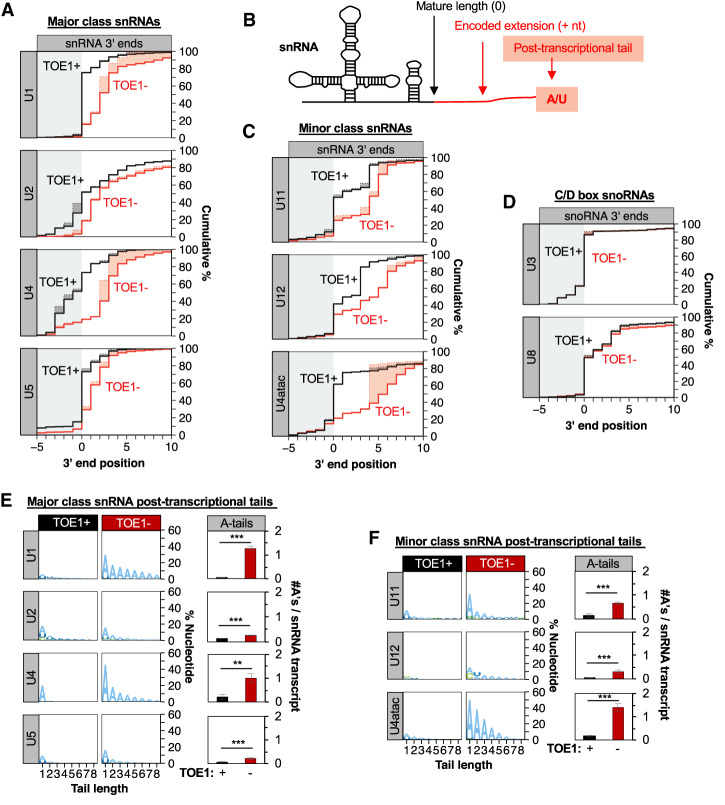
TOE1 processes RNA polymerase II transcribed snRNAs of the major and minor spliceosome. (*A*) Cumulative plots of nascent major class snRNA 3′ end positions from degron cells expressing (black line, TOE1^+^) or depleted for (red line, TOE1^−^) TOE1. snRNA 3′ ends were identified by RNA sequencing of nascent RNA isolated by metabolic labeling with 5-ethynyl uridine (5-EU) followed by purification using Click-iT technology. Position “0” refers to the mature 3′ end of snRNAs indicated by the border between gray and white backgrounds. Solid lines represent actual 3′ end positions of snRNAs including any posttranscriptional nucleotides, while dotted lines represent the predicted 3′ end of genome-encoded sequences with posttranscriptionally added nucleotides indicated by the shading between the lines. Only reads terminating at or downstream of position −5 are represented. Averages of at least three independent experiments are plotted for each snRNA. (*B*) Schematic of a U1 snRNA processing intermediate with the mature snRNA portion shown in black, a genome-encoded 3′ extension in red and posttranscriptionally added nucleotides are red with red shading. (*C*) Cumulative plots of nascent minor class snRNA 3′ end positions ±TOE1 as in *A*. (*D*) Cumulative plots of nascent C/D-box snoRNA 3′ end positions ±TOE1 as in *A*. (*E*, *left*) Sequence logo plots representing the percent of major class snRNAs with posttranscriptionally added nucleotides ±TOE1, broken down by nucleotide composition. Tail length refers to the number of posttranscriptional nucleotides added (up to eight shown). (*Right*) Average number of posttranscriptional adenosines per nascent major snRNA transcript when TOE1 is present (black) or depleted (red). (*F*) Sequence logo plots for minor class snRNA posttranscriptional nucleotides (*left*) and average number of posttranscriptional adenosines (*right*) as in *E*. Error bars indicate standard error of the mean (SEM) from at least three independent experiments. *P*-values (Student's two-tailed *t*-test): (**) *P* < 0.05; (***) *P* < 0.01. See also Supplemental Figure S1.

To investigate the importance of TOE1 in snRNA biogenesis we sought to generate cell lines with minimal TOE1 activity. Our past unsuccessful attempts at generating viable cell lines and mice deleted for the *TOE1* gene suggested that TOE1 is an essential protein (Lardelli et al. 2017). We therefore generated a human Flp-In T-Rex 293 cell line in which endogenous *TOE1* is knocked out and complemented by degron-tagged exogenous *TOE1* under the control of a doxycyline-inducible promoter. The degron-tagged TOE1 protein can be efficiently depleted by treatment of cells with auxin and is fully functional in U1 snRNA 3′ end processing (Supplemental Fig. S1A–C). To test the effect of TOE1 on the processing of snRNAs of the major and minor spliceosomes we performed 3′ end sequencing of nascent snRNAs isolated from TOE1 degron cells expressing (TOE1^+^) or depleted of (TOE1^−^) TOE1. The depletion of TOE1 resulted in an increased fraction of extended major and minor class snRNAs (Fig. 1A–C), revealing that TOE1 participates in 3′ end processing of all RNA polymerase II transcribed snRNAs of the major and minor spliceosomes. In contrast, TOE1 was not limiting for the 3′ end processing of U3 or U8 snoRNAs, which served as negative controls (Fig. 1D).

Analysis of the nucleotide composition of snRNA 3′ ends revealed that in addition to being incompletely processed, all tested snRNAs accumulated posttranscriptionally added adenosines in the absence of TOE1 (Fig. 1E,F). This was particularly notable for U1, U4, and U4atac snRNAs but also significant for all other tested snRNAs. In contrast to adenosines, levels of posttranscriptionally added uridines did not generally increase upon TOE1 depletion (Supplemental Fig. S1D). These observations demonstrate that TOE1 promotes the trimming of posttranscriptionally added adenosines and genome-encoded 3′ end tails of all RNA polymerase II transcribed snRNAs of the major and minor spliceosome.

### snRNAs are trimmed and tailed during multiple steps of processing

**Figure 2.**
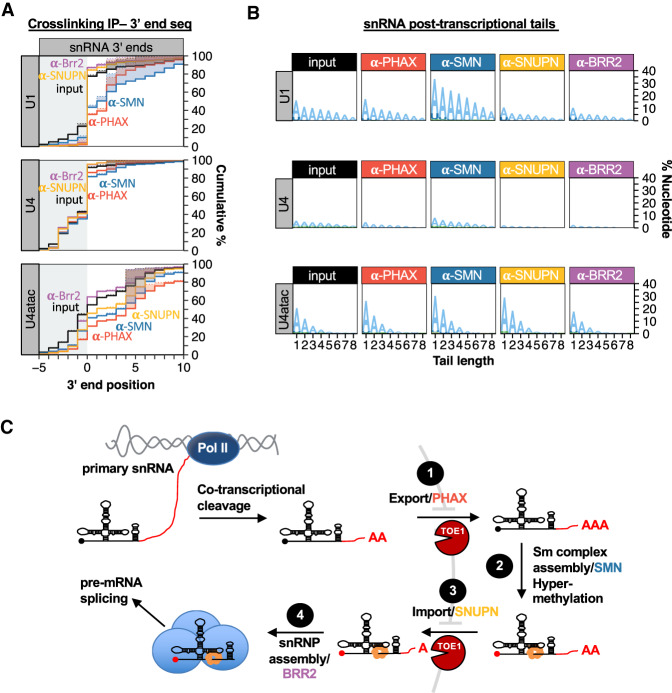
snRNAs are tailed and trimmed at early and late steps of biogenesis. (*A*) Cumulative plots of 3′ end positions for snRNAs associated with snRNA biogenesis factors PHAX (red), SMN (blue), SNUPN (yellow), and BRR2 (purple), monitored by cross-linking and immunoprecipitation followed by snRNA 3′ end sequencing. Input samples are shown in black. For U4 snRNA, BRR2-associated 3′ ends are almost indistinguishable from those associated with SNUPN. Only reads terminating at or downstream from position −5 are represented. The average of three independent experiments is plotted for each snRNA. (*B*) Sequence logo plots representing the percent of biogenesis factor-associated snRNAs that contain posttranscriptionally added nucleotides in the presence or absence of TOE1, broken down by nucleotide composition. Averages of three independent experiments are plotted for each condition. (*C*) Schematic of snRNA biogenesis showing dynamic posttranscriptional tailing and trimming occurring at early and late steps. See also Supplemental Figure S2.

To determine when during snRNA biogenesis 3′ end adenylation and trimming occurs we analyzed the 3′ ends of snRNAs associated with transient-acting snRNA biogenesis factors using formaldehyde cross-linking followed by immunoprecipitation (IP) and snRNA 3′ end sequencing (Fig. 2A,B; Supplemental Fig. S2). We focused on U1, U4, and U4atac snRNAs since these were among the snRNAs most affected by TOE1 depletion. Surprisingly, a large fraction of the population of U1 snRNA associated with the export factor PHAX and the Sm complex assembly factor SMN was mature length at the 3′ end (Fig. 2A), suggesting that a substantial fraction of the U1 snRNA pool is fully 3′ end processed at earlier stages of biogenesis than previously thought. A similar distribution of 3′ ends was seen for PHAX-associated U1 snRNAs in HeLa cells (Supplemental Fig. S2C,D). The U1 snRNA populations associated with the import factor SNUPN and the RNA helicase BRR2, which is a member of the U4/U6*U5 snRNP and the fully assembled spliceosome, were almost fully processed at the 3′ end and indistinguishable from the steady state U1 snRNA pool (Fig. 2A). This suggests that a second stage of 3′ end maturation takes place upon or after association of U1 snRNA with nuclear import machinery, and is consistent with previous observations of snRNA processing upon nuclear import in *Xenopus* oocytes (Yang et al. 1992). The 3′ end processing profile of U4 snRNA was similar to that of U1 snRNA but with an even greater fraction of fully mature snRNA associated with PHAX and SMN. The 3′ end of U4atac is more heterogeneous than that of U1 and U4 snRNAs but, as for U1 and U4 snRNAs, the U4atac snRNA pool is partially processed in association with PHAX and SMN and further processed with later stage factors (Fig. 2A; Supplemental Fig. S2D). Analysis of snRNA 3′ end nucleotide compositions revealed posttranscriptional tails primarily consisting of adenosines that for U1 and U4atac snRNAs were most prevalent in association with PHAX and SMN and for U4atac snRNA also with SNUPN (Fig. 2B; Supplemental Fig. S2B). These observations taken together suggest that a substantial fraction of U1, U4, and U4atac snRNAs are fully processed at the 3′ end prior to or during their association with the nuclear export factor PHAX with the remainder being processed upon or after association with nuclear import machinery (Fig. 2C). Furthermore, a dynamic process of 3′ end adenylation and deadenylation takes place during snRNA biogenesis.

### TOE1 initiates snRNA processing early in biogenesis

**Figure 3.**
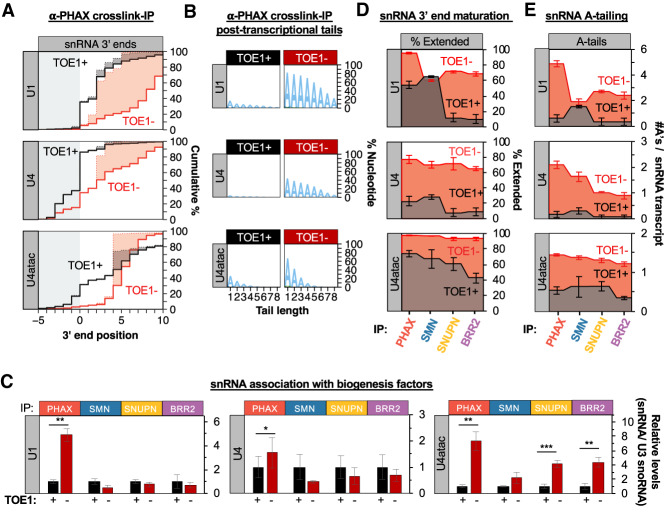
TOE1 depletion causes accumulation of unprocessed adenylated snRNAs with PHAX. (*A*) Cumulative plots of 3′ end positions of U1, U4, and U4atac snRNAs associated with PHAX in the presence (black) or absence (red) of TOE1, monitored by cross-linking and immunoprecipitation followed by snRNA 3′ end sequencing. (*B*) Sequence logo plots representing the percent of U1, U4, and U4atac snRNAs associated with PHAX that have posttranscriptional added nucleotides ±TOE1, broken down by nucleotide composition. (*C*) Relative levels of U1, U4, and U4atac snRNAs associated with biogenesis factors when TOE1 is present (black) or depleted (red) as measured by RT-qPCR assays normalized to the TOE1 nontarget control U3 snoRNA, with averages of normalized U1, U4, and U4atac snRNA levels when TOE1 is present set to 1. Error bars indicate SEM from three independent experiments. *P*-values (Student's two-tailed *t*-test): (*) *P* < 0.1; (**) *P* < 0.05; (***) *P* < 0.01. (*D*) Step plots showing the percentage of biogenesis factor-associated U1, U4, and U4atac snRNAs that are 3′ end extended in the presence (black) or absence (red) of TOE1 as monitored by cross-linking/immunoprecipitation and 3′ end sequencing. The average of three experiments is represented and SEM is represented by error bars. (*E*) Step plots representing the average number of posttranscriptional adenosines per snRNA transcript associated with snRNA biogenesis factors when TOE1 is present (black) or depleted (red). The average of three experiments is represented and SEM is represented by error bars. See also Supplemental Figure S3.

To test the importance of TOE1 for the early and late snRNA 3′ end maturation events, we monitored the effect of TOE1 depletion on the 3′ end processing and levels of U1, U4, and U4atac snRNAs associated with snRNA biogenesis factors. Cross-linking followed by IP and 3′ end sequencing revealed that the pools of PHAX-associated U1, U4, and U4atac snRNAs were remarkably less mature and more adenylated as a result of TOE1 depletion (Fig. 3A,B; Supplemental Fig. S3A–C). Moreover, TOE1 depletion resulted in a dramatic increase in the level of association of U1 and U4atac snRNAs with PHAX (Fig. 3C; Supplemental Fig. S3D) without remarkably affecting the nascent accumulation of these snRNAs (Supplemental Fig. S3E). U4 snRNA association with PHAX also increased upon TOE1 depletion albeit at a more modest level than observed for U1 and U4atac snRNAs (Fig. 3C; Supplemental Fig. S3D). Later stage biogenesis factor-associated snRNAs were also all less processed and more adenylated as a result of TOE1 depletion (Fig. 3D,E; Supplemental Fig. S3A,B) but only U4atac was observed to increase in levels of association with late stage factors (Fig. 3C). These observations demonstrate a role for TOE1 in trimming snRNA 3′ ends at both early and late steps of snRNA biogenesis. Furthermore, depletion of TOE1 perturbs the flux through the snRNA biogenesis pathway for each of the tested snRNAs, particularly at the export step as evidenced by their increased accumulation with PHAX.

### snRNAs are targets of the nuclear exosome in the absence of TOE1

**Figure 4.**
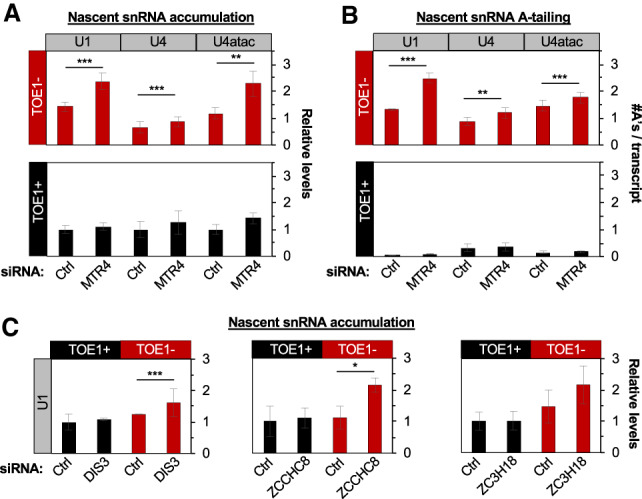
snRNAs become targets of the nuclear exosome in the absence of TOE1. (*A*) Relative levels of nascent snRNAs upon control (Ctrl) or MTR4 siRNA-mediated depletion in the absence (TOE1^−^) or presence (TOE1^+^) of TOE1. snRNA levels were measured by RT-qPCR and normalized to average snRNA levels in corresponding Ctrl siRNA/TOE1^+^ conditions (set to 1) with average levels of 7SK and mitochondrial 12S control RNAs serving as internal RT-qPCR normalization controls. (*B*) Average number of adenosines per snRNA transcript upon Ctrl or MTR4 siRNA-mediated depletion in the presence (TOE1^+^) or absence (TOE1^−^) of TOE1, monitored by 3′ end sequencing of nascent snRNAs. (*C*) Relative levels of U1 snRNA upon treatment with control siRNA (Ctrl) or siRNAs targeting nuclear exosome associated components, DIS3, ZCCHC8, and ZC3H18 compared with 7SK RNA and normalized to average snRNA levels in corresponding Ctrl siRNA/TOE1^+^ conditions. Error bars indicate SEM from at least three independent experiments for *A* and *B* and ZC3H18 in *C* and two independent experiments for DIS3 and ZCCHC8 in *C*. *P*-values (Student's two-tailed *t*-test): (*) *P* < 0.1; (**) *P* < 0.05; (***) *P* < 0.01. See also Supplemental Figure S4.

The nuclear exosome is known to target oligo-adenylated noncoding RNAs for degradation (LaCava et al. 2005; Vaňáčová et al. 2005; Wyers et al. 2005). Given the accumulation of oligo(A) tails and enhanced association of U1, U4, and U4atac snRNAs with PHAX upon TOE1 depletion, we wondered whether these snRNAs become targets of the nuclear exosome when TOE1 is absent. To test this idea, we monitored the effect of perturbing the nuclear exosome. The activity of the human nuclear exosome relies on one of several cofactor adapter complexes, all of which depend on the RNA helicase MTR4 (Schmid and Jensen 2018). When MTR4 was depleted in cells also depleted for TOE1 nascent U1 and U4atac snRNAs, and to a less extent U4 snRNA, were observed to increase in levels (Fig. 4A; Supplemental Fig. S4). This increase in levels was accompanied by increased accumulation of snRNA oligo(A) tails (Fig. 4B). In contrast, when TOE1 was present, cells depleted of MTR4 showed no significant increase in either the accumulation or adenylation of nascent snRNAs (Fig. 4A,B, bottom panels). Taken together, this suggests that snRNAs become adenylated and targeted by the nuclear exosome when TOE1 is absent, most evident for U1 and U4atac snRNAs. Depletion of other nuclear exosome factors, the nuclear exosome-associated exonuclease DIS3, the zinc finger protein ZCCHC8 of the nuclear exosome adapter NEXT complex, and the cap-binding complex (CBC)-associated exosome adaptor Zinc finger protein ZC3H18 also resulted in increased accumulation of nascent U1 snRNA when TOE1 was codepleted, confirming observations for MTR4 (Fig. 4C). These observations suggest that in addition to processing oligo(A) tails and 3′ ends of nascent U1, U4, and U4atac snRNAs, TOE1 prevents the targeting of nascent U1 and U4atac snRNAs, and to less of an extent U4 snRNA, by the nuclear exosome. Notably U1 and U4atac snRNAs, the snRNAs most affected by the nuclear exosome upon TOE1 depletion, were also the snRNAs most increased in PHAX accumulation (Fig. 3C), indicating a link between defective nuclear export and targeting by the nuclear exosome.

### TOE1 selectively processes regular U1 snRNA over unstable U1 snRNA variants

**Figure 5.**
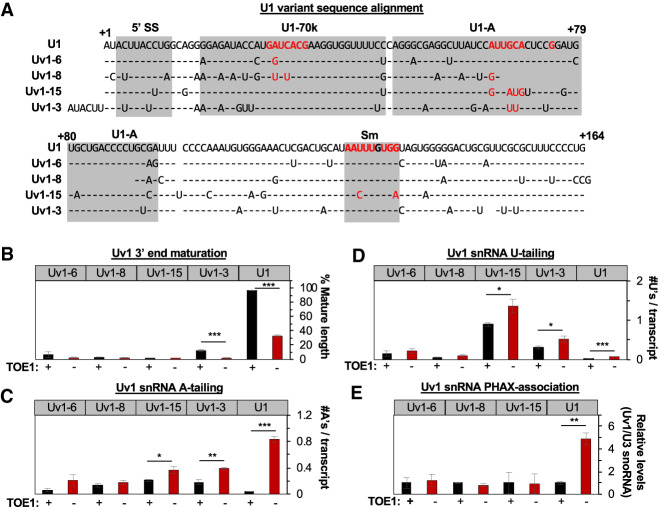
TOE1 selectively processes regular U1 snRNA over U1 snRNA variants. (*A*) Sequence alignment of U1 variant snRNAs with regular U1 snRNA based on O'Reilly et al. (2013). Gray boxes indicate RNA or protein interaction interfaces with critical protein-binding nucleotides highlighted in red (Kondo et al. 2015). (*B*) Percentage of mature length U1 variant snRNAs as monitored by 3′ end sequencing of RNA harvested at steady state when TOE1 is present (black) or depleted (red). (*C*) Average number of adenosines added per U1 variant snRNA transcript as monitored by 3′ end sequencing ±TOE1. (*D*) Average number of uridines added per U1 variant snRNA transcript as monitored by 3′ end sequencing ±TOE1. (*E*) Relative levels of association of U1 variants with PHAX when TOE1 is present (black) or depleted (red) as measured by RT-qPCR assays normalized to the TOE1 nontarget control U3 snoRNA, with averages of normalized U1 variant snRNA levels when TOE1 is present set to 1. Error bars indicate SEM from three independent experiments. *P*-values (Student's two-tailed *t*-test): (*) *P* < 0.1; (**) *P* < 0.05; (***) *P* < 0.01. See also Supplemental Figure S5.

We considered the possibility that the competing activities of TOE1 and the nuclear exosome at snRNA 3′ ends serve a function in quality control, and therefore turned to investigate U1 snRNA variants. We selected four U1 variant snRNAs, Uv1-3, Uv1-6, Uv1-8, and Uv1-15 (Fig. 5A) because we had previously been able to detect these in our sequencing assays at ∼0.01%–0.1% of regular U1 snRNA in Flp-In T-Rex 293 cells (Lardelli et al. 2017), which made it possible to monitor their 3′ ends. Each of these U1 variant snRNAs were notably unprocessed at the 3′ end as compared with regular U1 snRNA (Fig. 5B; Supplemental Fig. S5A). Depletion of TOE1 had little (Uv1-3) to no (Uv1-6, Uv1-8, and Uv1-15) effect on the accumulation of mature U1 variant snRNAs (Fig. 5B), although some nibbling of U1 variant snRNA 3′ ends by TOE1 could be detected to varying degrees (Supplemental Fig. S5A). The extent and composition of posttranscriptional tailing varied between the tested U1 variant snRNAs, but, in general, they accumulated both A and U tails at higher levels than observed for regular U1 snRNA when TOE1 was present (Fig. 5C,D; Supplemental Fig. S5B). Depletion of TOE1 showed no significant effect on the accumulation of posttranscriptional tails of Uv1-6 and Uv1-8 snRNAs, and a modest increase in adenylation and uridylation of Uv1-3 and Uv1-15 snRNAs as compared with the much stronger increase in adenylation observed for regular U1 snRNA (Fig. 5C,D; Supplemental Fig. S5B). In striking contrast to regular U1 snRNA, the association of Uv1-6, U1v-8, and U1v-15 snRNAs with PHAX was entirely unaffected by TOE1 depletion (Fig. 5E; Supplemental Fig. S5C,D); we were unable to establish a qPCR assay with sufficient specificity to test this for Uv1-3. Taken together, these observations demonstrate that tested U1 variant snRNAs are poor substrates for TOE1, ranging from nontargets to minor targets as compared with regular U1 snRNA.

### U1 variant snRNAs are targets of the nuclear exosome

**Figure 6.**
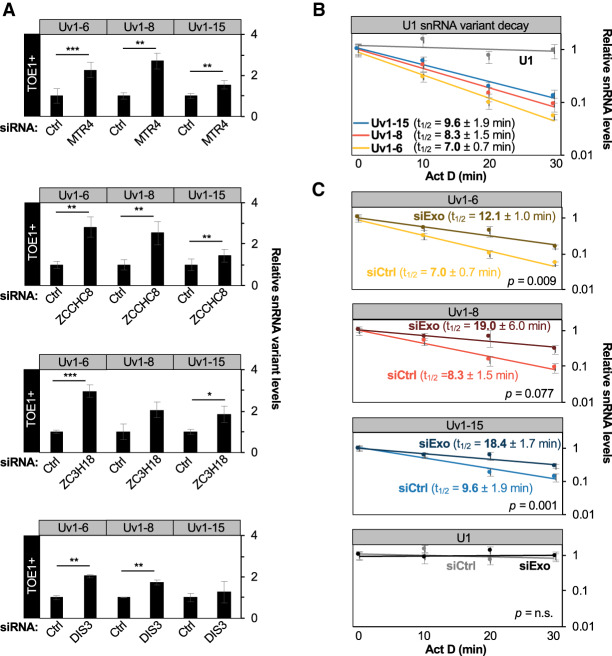
U1 variant snRNAs are targets of the nuclear exosome. (*A*) Relative levels of U1 variant snRNAs after siRNA-mediated depletion of MTR4, ZCCHC8, ZC3H18, and DIS3 as measured by RT-qPCR from total RNA and normalized to average values for negative control (Ctrl) Luciferase siRNA-treated samples. Averages of mitochondrial 12S and 7SK RNA levels served as internal normalization controls. (*B*) SnRNA turnover assays monitoring the fraction of U1 variant snRNAs remaining after actinomycin D-mediated transcription shut-off compared with the 0 time point (no actinomycin D) as measured by RT-qPCR. Shown half-lives (*t*_1/2_) were calculated after normalization of U1 variant levels to the average of 7SK and 12S RNA levels. (*C*) SnRNA turnover assays for U1 variant snRNAs after siRNA-mediated depletion of MTR4 and ZCCHC8 (siExo) as compared with a control siRNA (siCtrl) with normalization as in *B*. Error bars indicate SEM from at least three independent experiments. *P*-values (Student's two-tailed *t*-test): (*) *P* < 0.1; (**) *P* < 0.05; (***) *P* < 0.01. See also Supplemental Figure S6.

To test whether U1 variant snRNAs are targeted by the nuclear exosome, we depleted nuclear exosome cofactors and monitored the accumulation of Uv1-6, Uv1-8, and Uv1-15 snRNAs. Contrasting regular U1 snRNA, which was only affected by the nuclear exosome when TOE1 was depleted (Fig. 4), depletion of nuclear exosome factors MTR4, ZCCHC8, ZC3H18, and DIS3 resulted in increased accumulation of Uv1-6, Uv1-8, and Uv1-15 snRNAs even in the presence of TOE1 (Fig. 6A), an effect that was further enhanced upon codepletion of two exosome cofactors, MTR4 and ZCCHC8 (Supplemental Fig. S6A,B). Unlike regular U1 snRNA, codepletion of TOE1 did not further increase the accumulation of the U1 variant snRNAs (Supplemental Fig. S6C). To more directly test the effect of the nuclear exosome on variant U1 snRNA stability we next monitored the degradation of variant U1 snRNAs using actinomycin D transcription shut-off assays. Uv1-6, Uv1-8, and Uv1-15 snRNAs all displayed remarkably short half-lives of <10 min (Fig. 6B). Codepletion of MTR4 and ZCCHC8 resulted in stabilization of all three U1 snRNA variants (Fig. 6C). These observations demonstrate that in contrast to regular snRNAs, which are efficiently processed by TOE1 to promote snRNA biogenesis and exclude nuclear exosome degradation, Uv1-6, Uv1-8, and Uv1-15 variant snRNAs are largely unprocessed by TOE1 and are instead targeted for decay by the nuclear exosome.

## Discussion

**Figure 7.**
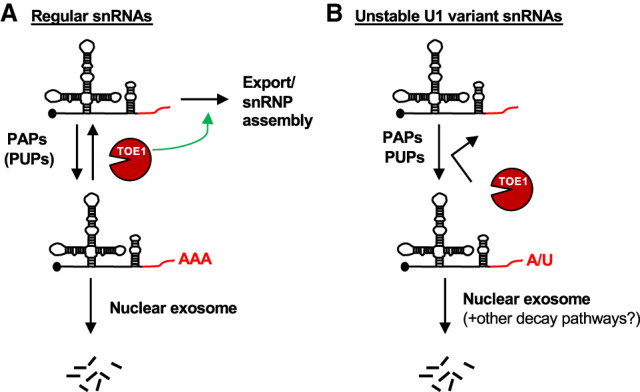
TOE1 selectively rescues regular snRNAs from nuclear decay and promotes their maturation. (*A*) TOE1 opposes adenylation and processes 3′ tails of regular snRNAs, thus rescuing them from decay by the nuclear exosome and promoting their progression through snRNA biogenesis. (*B*) Variant U1 snRNAs are poorly processed by TOE1 and instead are substrates for degradation.

Enzymes acting on RNA 3′ ends can promote the maturation or degradation of RNAs, but how these enzymes compete to ultimately control the fate of cellular RNAs remains an outstanding question. Here, we present evidence that the human DEDD-deadenylase TOE1 promotes the maturation of RNA polymerase II transcribed snRNAs in competition with degradation by the nuclear exosome in a process that helps differentiate the fates of regular spliceosomal snRNAs from genome-encoded unstable U1 snRNA variants (Fig. 7). Indeed, all regular RNA polymerase II transcribed snRNAs of the major and minor spliceosomes accumulated with unprocessed and adenylated 3′ ends upon TOE1 depletion (Fig. 1), while all tested U1 snRNA variants accumulated with 3′ end extensions regardless of the presence of TOE1 (Fig. 5; Supplemental Fig. S5). Furthermore, TOE1 depletion led to increased accumulation of extended and adenylated snRNAs with the early acting snRNA biogenesis export adapter PHAX suggesting a delay at the export step (Fig. 3), while PHAX association of variant U1 snRNAs remained unaffected (Fig. 5). Finally, depletion of nuclear exosome components and cofactors led to increased accumulation and adenylation of nascent regular snRNAs only when TOE1 was codepleted (Fig. 4), whereas the levels and half-lives of variant U1 snRNAs increased upon depletion of exosome factors regardless of the presence of TOE1 (Fig. 6). Our findings place TOE1 at the center of a quality control pathway that discriminates regular snRNAs from unstable snRNA variants, promoting the biogenesis of the former while leaving the latter unprocessed and exposed to degradation (Fig. 7).

A surprising finding from our study is that TOE1 initiates trimming of snRNAs early in biogenesis, before or during association with the export factor PHAX, in addition to further trimming upon snRNA reimport into the nucleus first reported >25 yr ago (Yang et al. 1992). The degree to which individual snRNAs are trimmed in early biogenesis appears to be specific to the snRNA species (Figs. 2A, 3A,D). The observation that, in the presence of TOE1, there is little to no increase in the fraction of mature length snRNAs associated with SMN as compared with PHAX suggests that snRNAs are not trimmed again until the import step, where further trimming is evidenced by an increased fraction of mature U1 and U4 snRNAs associated with SNUPN and BRR2, and U4atac with BRR2 (Fig. 2). An alternative explanation for the increased fraction of mature length snRNAs associating with later biogenesis factors is that unprocessed snRNAs are degraded rather than 3′ end processed and/or that late-stage factors show specificity toward 3′ end processed snRNAs. However, taken together with the previous observations of snRNA processing after nuclear import (Yang et al. 1992) and the observation that TOE1 localizes to the nucleus and concentrates in Cajal bodies (Wagner et al. 2007; Fong et al. 2013), the most parsimonious interpretation is that TOE1 processes snRNA 3′ ends during both of the nuclear stages of snRNA biogenesis, initiating the processing of nascent snRNA molecules prior to or upon nuclear export and finalizing maturation of remaining unprocessed molecules after nuclear import.

Our observations suggest that TOE1 promotes the biogenesis of regular snRNAs by at least two mechanisms that could be related to one another. First, the increased association of U1 and U4atac, and to a lesser extent U4, snRNAs with PHAX observed in the absence of TOE1 (Fig. 3C) suggests that TOE1 is important for promoting the normal progression of snRNAs through the nuclear export step. Increased association of U4atac snRNA also with downstream biogenesis factors upon TOE1 depletion (Fig. 3C) suggests that for this particular snRNA, additional biogenesis steps are sensitive to 3′ end processing as well. Second, the accumulation of snRNAs upon depletion of nuclear exosome factors in the absence of TOE1 (Fig. 4) suggests that TOE1 protects snRNAs from degradation by the nuclear exosome. While it is possible that unprocessed snRNAs can be targeted by the nuclear exosome later in biogenesis after nuclear reimport, it is also possible that nuclear export and decay are linked. For example, a delay in snRNA nuclear export upon TOE1 depletion could result in increased exposure to the nuclear exosome. The observation that U4 snRNA, which is less affected than U1 and U4atac snRNAs in PHAX association upon TOE1 depletion, is also less sensitive to the exosome (Figs. 3C, 4A) is consistent with this idea and suggests that 3′ end trimming is less critical for U4 than for U1 and U4atac snRNA biogenesis. TOE1 could affect these processes by directly interacting or competing with biogenesis or degradation factors or, more likely, through its trimming of snRNA 3′ ends. Consistent with the latter, PHAX-associated snRNAs are the most highly adenylated (Fig. 3E), suggesting that adenylation occurs primarily early in snRNA processing, and RNA oligoadenylation is a well-documented targeting mechanism for the nuclear exosome (LaCava et al. 2005; Vaňáčová et al. 2005; Wyers et al. 2005; Meola et al. 2016; Shukla et al. 2016; Shukla and Parker 2017). It remains to be determined whether oligo(A) tailing can also influence RNA nuclear export.

A key finding of our study is that TOE1 distinguishes regular snRNAs from tested unstable U1 snRNA variants promoting biogenesis only of the former. How TOE1 distinguishes regular from variant snRNAs remains an outstanding question. It is possible that TOE1 intrinsically recognizes specific sequence features or structures of snRNAs such as the 5′ cap or the Sm-binding site that are in common between all regular RNA polymerase II transcribed snRNAs. This, however, seems unlikely given that three of the four tested U1 snRNA variants have intact Sm-binding site sequences (Fig. 5A) and all are predicted to have 5′ caps. Alternatively, TOE1 may associate with snRNP proteins or biogenesis factors that assemble only with regular snRNAs. Consistent with this idea, TOE1 displays RNase-resistant association with several snRNP factors (Fong et al. 2013; Lardelli et al. 2017) and each of the tested U1 variant snRNAs have sequence variations in key U1 snRNA protein binding sites (Fig. 5A). Finally, it cannot be ruled out that TOE1 is recruited cotranscriptionally by a mechanism that is specific to regular snRNAs; however, we observed no evidence for association between TOE1 and transcription machinery or the Integrator complex in our previous coimmunoprecipitation experiments (Lardelli et al. 2017).

An important question is how defective 3′ end processing and posttranscriptional tailing of U1 snRNA variants relate to their degradation. An obvious possibility is that unprocessed 3′ end extensions serve as entry points for degradation machinery including the nuclear exosome and possibly other degradative enzymes. Interestingly, posttranscriptional tailing of the tested U1 snRNA variants was generally more prevalent than that of regular U1 snRNA at steady state (Fig. 5C,D), but the nucleotide compositions of the tails differed between variants (Supplemental Fig. S5B). Uv1-15 snRNA in particular was highly uridylated as compared with regular U1 snRNA and other U1 snRNA variants, which showed a more even distribution between adenylation and uridylation (Fig. 5C,D; Supplemental Fig. S5B). Uv1-15 differs from the other U1 variant snRNAs as the only variant with a nucleotide variation in the Sm-binding site, a type of change that has previously been shown to trigger snRNA degradation (Shukla and Parker 2014). Thus, snRNA variants left unprocessed by TOE1 may be targeted by different tailing enzymes, and potentially, by additional nucleases beyond the nuclear exosome depending on their specific nucleotide variations.

It remains to be determined how general the snRNA 3′ end quality control mechanism described here is in the repression of the hundreds of snRNA variants encoded in the mammalian genome. Some human U1 snRNA variants are identical (e.g., Uv1-18) or near identical (e.g., Uv1-7 and Uv1-9) in sequence to regular U1 snRNA and likely retain normal snRNA processing and function, while other variants may never be transcribed in the first place (O'Reilly et al. 2013). Either way, this 3′ end quality control mechanism could represent a widespread pathway of quality control for noncoding RNAs even beyond snRNAs given the large number of noncoding RNAs now known to be processed by the DEDD family of deadenylases, PARN, TOE1, and PNLDC1 (Berndt et al. 2012; Lardelli et al. 2017; Shukla and Parker 2017; Zhang et al. 2017; Son et al. 2018). Consistent with this idea, telomerase and Y RNAs are destabilized in the absence of PARN (Moon et al. 2015; Shukla and Parker 2017), which in the case of telomerase RNA likely contributes to symptoms of dyskeratosis congenita patients with genetic mutations in the *PARN* gene.

Whether defects in snRNA biogenesis contribute to symptoms of pontocerebellar hypoplasia (PCH) 7, which is caused by *TOE1* mutation (Lardelli et al. 2017), is unknown; however, consistent with this idea, mutation of other snRNA biogenesis factors and mutation of snRNAs themselves are causal to neurodevelopmental disorders that have overlapping symptoms with PCH, including mutation of *SMN* in spinal muscular atrophy (Lefebvre et al. 1995), mutation of *INTS1* and *INTS8* of the integrator complex in neurodevelopment (Oegema et al. 2017) and mutation of U4atac snRNA in microcephalic osteodysplastic primordial dwarfism type 1/Taybi-Linder syndrome (Abdel-Salam et al. 2011; He et al. 2011). Additionally, mutations in exosome core subunits and in *CLP1*, which has been implicated in snRNA 3′ end processing (Hallais et al. 2013), have shown to be causal to other subtypes of PCH (Wan et al. 2012; Boczonadi et al. 2014; Schaffer et al. 2014), consistent with the idea that maintaining the balance of enzyme activity at the 3′ end of RNA is crucial to proper neurological function. TOE1 appears to be uniquely limiting for the processing of snRNAs but acts redundantly with PARN on a number of other noncoding RNAs (Son et al. 2018; Deng et al. 2019). Thus, it is possible that defects in the processing of RNAs other than snRNAs contributes to PCH7; for example, if TOE1 is limiting for certain processing events specifically in affected tissues. In either case, a picture is developing where DEDD-deadenylases are central to the proper maturation and maintenance of levels of a wide variety of noncoding RNAs and that defects in these pathways lead to devastating human disorders.

## Materials and methods

### Stable TOE1 degron cell line generation

Gibson assembly (New England Biolabs; NEB) was used to fuse one repeat of minimal auxin-inducible degron (mAID) fragment (Nishimura et al. 2009) synthesized as a gBlock gene fragment (Integrated DNA Technologies [IDT]) to the C terminus of TOE1 in the pcDNA5/FRT/TO-Flag-TOE1 construct (Lardelli et al. 2017) to generate pcDNA5/FRT/TO-Flag-TOE1-mAID, and stable HEK FLp-In T-REx-293 (Invitrogen) cell lines expressing Flag-TOE1-mAID under control of a tetracycline-inducible promoter were generated using this plasmid according to manufacturer's recommendations (Invitrogen). To generate cell lines expressing F-box protein osTIR1, the coding region of *TIR1* from *Oryza sativa* lacking a stop codon and with flanking *att*B recombination sites was synthesized as a gBlock gene fragment (IDT) and cloned into pDONR221 using the BP clonase Gateway reaction (Thermo Fisher), and then inserted into *att*R sites of pLEX307 that had been modified to include a C-terminal mKate2 tag to generate pLEX307-osTIR-mKate2 (the original pLEX_307 plasmid was a gift from David Root; Addgene plasmid 41392, http://n2t.net/addgene:41392, RRID:Addgene_41392). To generate TOE1-mAID expressing cell lines with stable osTIR1 expression, pLEX307-osTIR-mKate2 was linearized with *Not*I and transfected into the HEK FLp-In T-REx-293 Flag-TOE1-mAID cells. osTIR1-mKate2-expressing clones were manually picked following selection in medium containing 1 µg/mL puromycin for 10–14 d. Clones displaying auxin-induced depletion of Flag-TOE1-mAID as monitored by Western blotting were then transfected with pSpCase9(BB)-2A-GFP plasmids expressing guide (g)RNAs targeting the endogenous *TOE1* gene (see gRNA sequences in Supplemental Table S1; Ran et al. 2013). pSpCas9(BB)-2A-GFP (PX458) was a gift from Feng Zhang (Addgene plasmid 48138, http://n2t.net/addgene:48138, RRID:Addgene_48138). Cells were sorted for GFP expression 2 d after transfection and single colonies were picked, expanded, and tested for knockout of the endogenous *TOE1* gene with PCR using primers flanking the genomic region predicted to be excised (Supplemental Table S1). HEK FLp-In T-REx-293 Flag-TOE1-mAID/ΔTOE1 cell lines, subsequently named degron cells, were validated by Western blotting and RNA sequencing (Supplemental Fig. S1 and below). Mycoplasma testing was routinely performed and all cell lines were negative.

### Cell growth and depletions

All cells were maintained in Dulbecco's modified Eagle medium (DMEM; Gibco) with 10% fetal bovine serum (FBS; Gibco) and 1% penicillin/streptomycin (Gibco). Degron cells were depleted (TOE1^−^) of Flag-TOE1-mAID with 500 µM of the auxin hormone indole-3-acetic acid (IAA; Sigma) or induced (TOE1^+^) with 1 ng/mL doxycycline 48 h before harvest. Knockdowns in degron cells were performed with 20 nM small interfering (si)RNA targeting either luciferase (Ctrl) or exosome components (Supplemental Table S1) using siLentFect (Bio-Rad) transfection reagent according to manufacturer's recommendations at 72 and 24 h before harvest.

### Nascent RNA 3′ end sequencing and accumulation assays

Cells grown in six-well plates treated with 0.2 mM ethynyl uridine (Thermo Fisher) for 8 h were harvested in 1 mL of TRIzol (Thermo Fisher), depletions were performed as described above and total RNA was isolated according to the manufacturer's recommendation. RNA adapters containing barcodes and 10- to 11-nt random mers (AG10/AG11) were ligated to the 3′ ends of 2.5 µg of extracted RNA in a 10-µL reaction containing 1 µL of 10xT4 RNA ligase buffer (500 mM Tris-HCl, 100 mM MgCl_2_, 10 mM DTT at pH at 25°C), 0.2 mg/mL bovine serum albumin (BSA), 2 µM AG10 or AG11, 1 mM ATP, 10 U of T4 RNA ligase (NEB), and 40 units of RNaseOUT (Invitrogen) for 16 h at 16°C and extracted with phenol/chloroform/isoamyl alcohol as described previously (Lardelli et al. 2017). Half of the purified, ligated RNA underwent Click reaction to biotinylate EU-containing RNA with 0.25 mM biotin azide using Click-it nascent RNA capture kit (Thermo Fisher) per manufacturer's recommendations. One-half of the Click reaction RNA was purified with 12 µL Dynabeads MyOne strepavidin T1 beads per manufacturer's recommendations (Thermo Fisher) with the exception that after washes on-bead cDNA was generated using SuperScript III (SSRT III; Thermo Fisher) in a 20-µL reaction using 0.5 µM linker-specific primer AR-17 (primers supplied in Supplemental Table S1). For sequencing, snRNA cDNA 3′ ends were amplified in 16–25 cycles of polymerase chain reaction (PCR) with snRNA gene-specific forward primers (Supplemental Table S1) and AR-17 primer using Q5 DNA polymerase (NEB), and then eight cycles with primers D50x and D70x (Illumina; Supplemental Table S1). Libraries were purified, quantified, and sequenced, and mapped and analyzed using custom python scripts as previously described (Lardelli et al. 2017). Cumulative 3′ end position plots were generated using ggplot2 (Wickham 2009). Sequence logo plots were generated using ggseqlogo (Wagih 2017).

### Cross-linking and immunoprecipitation followed by 3′ end sequencing

Degron cells treated to induce or deplete Flag-TOE1-mAID as described above or HeLa cells treated with siCtrl as described above were cross-linked with 0.2% formaldehyde (from a 37% HCHO/10% methanol stock) for 10 min at room temperature. Cross-linking reactions were quenched by adding glycine (pH 7.0) to 0.25 M, incubating for 5 min at room temperature and washing three times with ice cold phosphate-buffered saline (PBS; 137 mM NaCl, 2.7 mM KCl, 8 mM Na_2_HPO_4_, 2 mM KH_2_PO_4_). Cell pellets were resuspended in 0.5 mL of RIPA buffer (50 mM Tris-HCl at pH 7.5, 1% Nonidet P-40, 0.5% sodium deoxycholate, 0.05% SDS, 1 mM EDTA, 150 mM NaCl, 2 µg/mL aprotinin and leupeptin, 1 mM phenylmethylsulfonyl fluoride [PMSF]) per 10-cm plate, and lysates were prepared as described previously (Niranjanakumari et al. 2002). For each immunoprecipitation (IP), 1.0–1.5 mg of lysate was used. Between 5 and 20 µg of polyclonal antibodies (15 µg of rabbit anti-PHAX from Bethyl, 5 µg of rabbit anti-SMN from MBL International, 20 µg of rabbit anti-Snurportin 1 from Sigma, 2 µg of rabbit anti-SNRNP200 from Bethyl) were precoupled with 30 µL Protein A Dynabeads (Thermo Fisher) per IP, nutated in 0.5% BSA at 4°C overnight, and washed three times in NET-2 (10 mM Tris-HCl at pH 7.5, 150 mM NaCl, 1 mM EDTA) and one time in RIPA buffer. IPs were carried out by mixing antibody-coupled Dynabeads with cell lysates for 90 min at room temperature, and then washed six times with denaturing wash buffer (50 mM Tris-HCl at pH 7.5, 1% NP-40, 1% sodium deoxycholate, 0.1% SDS, 1 mM EDTA, 500 µM NaCl, 2 M urea, 0.2 mM PMSF) for 10 min per wash nutating at room temperature. After the final wash, beads were washed three times in NET-2. On bead ligations of AG10 or AG11 to RNA were performed in 20-µL reactions (containing 2 µL 10xT4 RNA ligase buffer, 0.2 mg/mL BSA, 2 µM AG10 or AG11, 1 mM ATP, 20 U of T4 RNA ligase [NEB]) overnight with gentle shaking at 16°C. Cross-links were reversed for 45 min at 70°C and RNA was extracted using TRIzol. Purified, ligated RNA was used to make AR-17 primed cDNA using SSRTIII. For sequencing, 3′ ends were PCR amplified with AR-17 and gene-specific primers by Q5 DNA polymerase (NEB) as described above for 20–28 cycles and purified with Ampure beads (Beckman) before eight cycles of Q5 DNA polymerase PCR amplification with D50x and D70x primers. Libraries were sequenced as described previously (Lardelli et al. 2017).

### Variant-specific sequencing

Variant U1 snRNA 3′ ends were amplified for 3′ end sequencing with variant-specific primers (Supplemental Table S1) and AR-17 from cDNA made with AR-17-primed SSRTIII cDNA from linker ligated total RNA prepared using TRIzol without a nascent RNA capture step.

### Transcription shut-off assays

Cells were treated with siRNA 72 and 24 h as described above before transcription shut-off by the addition of 5 µg/mL actinomycin D to cells for 30, 20, or 10 min before harvest in TRIzol (Thermo Fisher). Total RNA was 3′ end-ligated (as described above) and cDNA synthesis was primed with AR17.

### Quantitative PCR

For relative quantification of nascent RNA, snRNA variant RNA, and immunopurified RNA, AR17-primed cDNA (as described above) was amplified using Fast SYBR Green master mix (Thermo Fisher) with snRNA/snoRNA-specific forward and reverse primers (Supplemental Table S1) on a StepOnePlus real-time instrument (Applied Biosystems). Relative levels were quantified using the ΔΔC_t_ method (Livak and Schmittgen 2001).

### Western blotting

Western blots were performed with rabbit polyclonal anti-Caf1z/TOE1 (Wagner et al. 2007) at 1:1000, rabbit polyclonal anti-Upf1 (Lykke-Andersen et al. 2000) at 1:1000, polyclonal rabbit anti-MTR4 (Abcam) at 1:1000, anti-mouse polyclonal anti-ZCCHC8 (Abcam) at 1:1000, rabbit polyclonal anti-ZC3H18 (Sigma) at 1:500, and rabbit polyclonal anti-DIS3 (Bethyl) at 1:1000, all in 5% nonfat milk in PBS with 0.1% Tween (PBST) overnight at 4°C. Secondary antibodies used were goat anti-rabbit IRDye 680RD (LI-COR) at 1:15,000, or HRP goat antimouse and HRP donkey antirabbit at 1:20,000 in 5% nonfat milk in PBST. Western blots were visualized using an Odyssey Fc imaging system (LI-COR).

### Data accessibility

RNA sequencing data have been deposited into the Gene Expression Omnibus (GEO) under accession number GSE141709.

## Supplementary Material

Supplemental Material
