# Supplementary material for: Competition between maturation and degradation drives human snRNA 3′ end quality control
Source: Genes Dev. 2020 Jul 1;34(13-14):989–1001. doi: 10.1101/gad.336891.120 (PMC7328512; doi:10.1101/gad.336891.120)
Supplement: Supplemental Material [file supp_34_13-14_989__index.html]

Competition between maturation and degradation drives human snRNA 3′ end quality control — Supplemental Material 

# Competition between maturation and degradation drives human snRNA 3′ end quality control

## Supplemental Material

- Supplemental\_Data.pdf
