# Supplementary material for: Competition between maturation and degradation drives human snRNA 3′ end quality control
Source: Genes Dev. 2020 Jul 1;34(13-14):989–1001. doi: 10.1101/gad.336891.120 (PMC7328512; doi:10.1101/gad.336891.120)
Supplement: Supplemental Material [file supp_gad.336891.120_Supplemental_Data.pdf]

Competition between end maturation and degradation drives human snRNA 3’ end quality control

Rea M. Lardelli and Jens Lykke-Andersen

Supplemental Material Titles

Supplemental Figure S1. Related to Figure 1. Characterization of TOE1-degron cell lines.

Supplemental Figure S2. Related to Figure 2. snRNAs are tailed and trimmed at early and late steps of snRNA biogenesis.

Supplemental Figure S3. Related to Figure 3. TOE1 depletion causes accumulation of unprocessed adenylated snRNAs with PHAX.

Supplemental Figure S4. Related to Figure 4. snRNAs become targets of the nuclear exosome in the absence of TOE1.

Supplemental Figure S5. Related to Figure 5. TOE1 selectively processes regular U1 snRNA over U1 snRNA variants.

Supplemental Figure S6. Related to Figure 6. U1 variant snRNAs are targets of the nuclear exosome.

Supplemental Table S1. List of DNA and RNA oligos used.

Supplemental Fig. S1

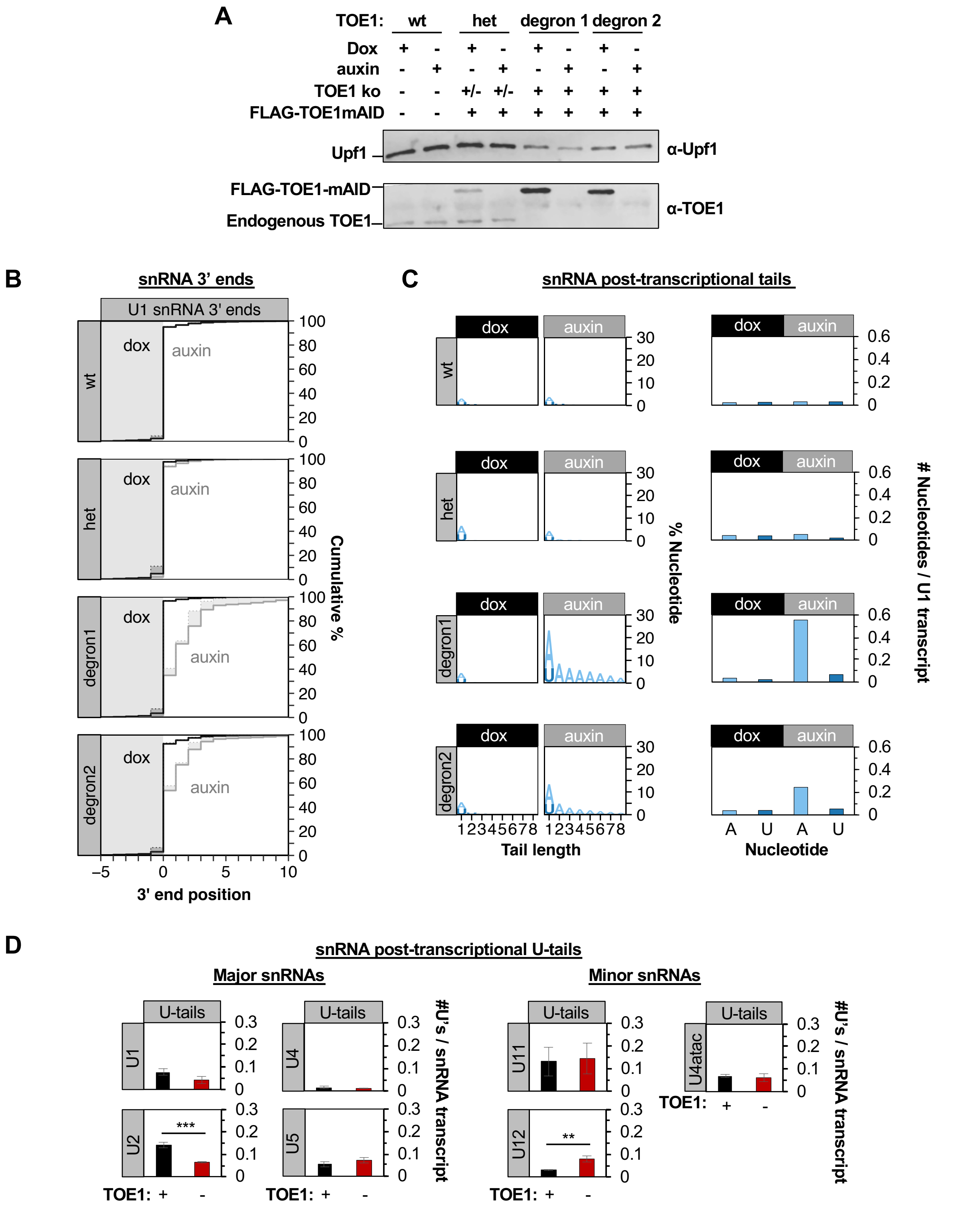

Supplemental Figure S1 related to Figure 1. Characterization of TOE1-degron cell lines. **(A)** Western blot analysis of endogenous TOE1 and FLAG-TOE1-mAID levels in TOE1-degron cell lines treated with doxycycline (Dox) or auxin for 48 hours. Western blotting for Upf1 served as a loading control. Wt: parental cell line; Het: cell line expressing both endogenous TOE1 and exogenous FLAG-TOE1mAID; Degron 1 and 2: cell lines expressing exogenous FLAG-TOE1mAID with endogenous *TOE1* knocked out. Auxin treatment resulted in levels of FLAG-TOE1mAID below the limit of detection. **(B)** Cumulative plot of 3' ends of U1 snRNA harvested at steady state from wt, het, degron 1 and 2 cell lines described in *(A),* treated with doxycycline (black line) or auxin (gray line) for 48 hours. Position '0' refers to the mature 3' end of snRNAs indicated by the border between gray and white backgrounds. Only reads terminating at or downstream of position -5 are represented. Solid lines represent actual 3' end positions of snRNAs including any post-transcriptional nucleotides, while dotted lines represent the predicted 3' end of genome-encoded sequences with post-transcriptionally added nucleotides indicated by the shading between the lines. **(C)** Left panels: Sequence logo plots representing the percent of U1 snRNA with post-transcriptionally added nucleotides, broken down by nucleotide composition, for each cell line treated with doxycycline (black) or auxin (gray) for 48 hours. Right panels: average number of post-transcriptional adenosines and uridines per U1 snRNA transcript monitored by snRNA 3' end sequencing from indicated cell lines treated with doxycycline or auxin as indicated. **(D)** Average number of post-transcriptional uridines per snRNA transcript monitored by snRNA 3' end sequencing when TOE1 is present (black) or depleted (red) from the degron 1 cell line. Error bars: SEM from at least three independent experiments and p-values (Student's two-tailed t-test) **:$p<0.05$; ***:$p<0.01$.

Supplemental Fig. S2

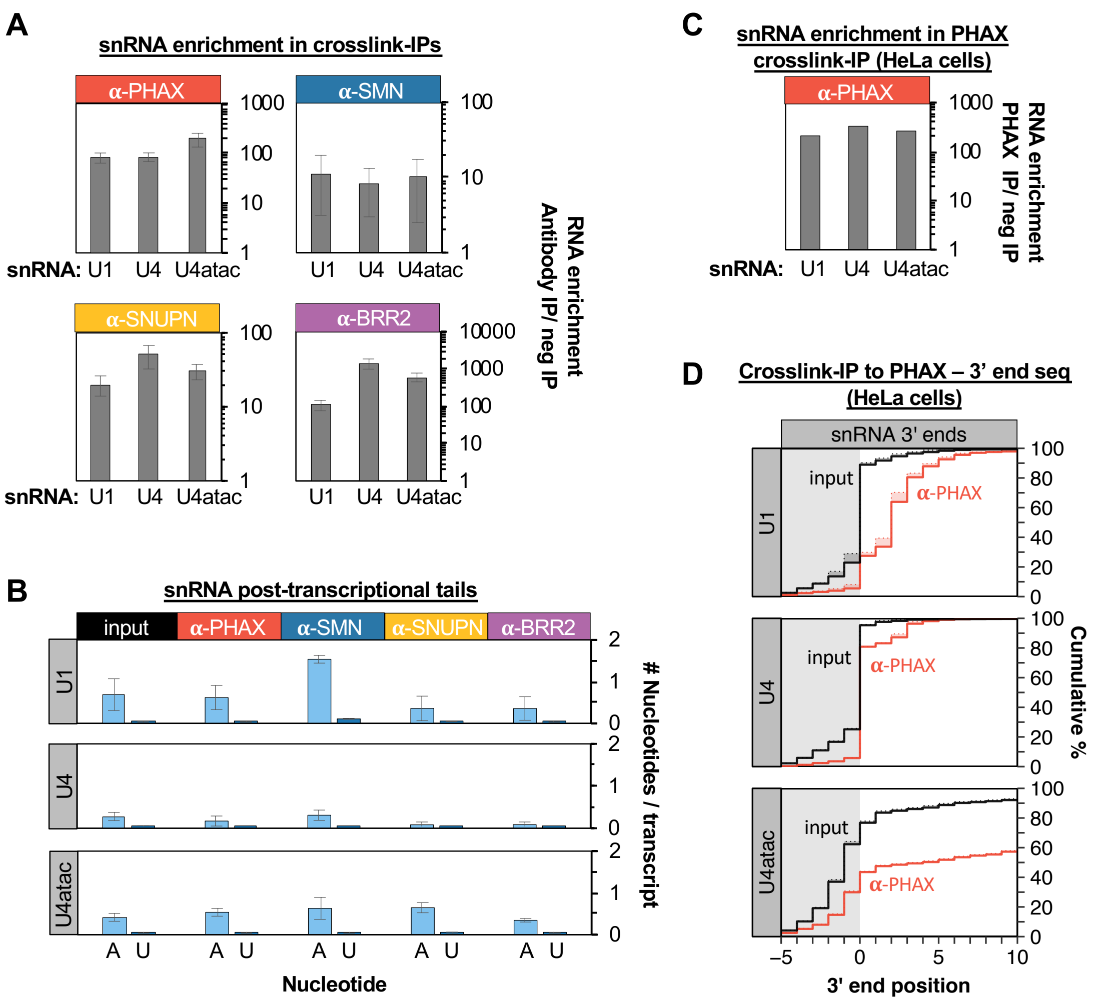

Supplemental Figure S2 related to Figure 2. snRNAs are tailed and trimmed at early and late steps of snRNA biogenesis. (A) Enrichment of U1, U4 and U4atac snRNAs in crosslinking and immunoprecipitation experiments with antibodies against PHAX, SMN, SNUPN, and BRR2 as measured by RT-qPCR and normalized to immunoprecipitation reactions with no antibody, with the latter condition set to 1. **(B)** Average number of post-transcriptionally added adenosines and uridines per snRNA transcript immunoprecipitated with indicated biogenesis factors. Input: snRNAs from cell lysates prior to immunoprecipitation. Error bars: SEM from at least three independent experiments. **(C)** Enrichment of U1, U4 and U4atac snRNAs in crosslinking and

immunoprecipitation experiments with antibodies against PHAX from HeLa cells as measured by RT-qPCR and normalized to immunoprecipitation reactions with no antibody, with the latter condition set to 1. **(D)** Cumulative plots of 3' end positions for snRNAs associated with snRNA biogenesis factor PHAX (red) from HeLa cells monitored by crosslinking and immunoprecipitation followed by snRNA 3' end sequencing. Input samples are shown in black. Only reads terminating at or downstream of position -5 are represented.

Supplemental Fig. S3

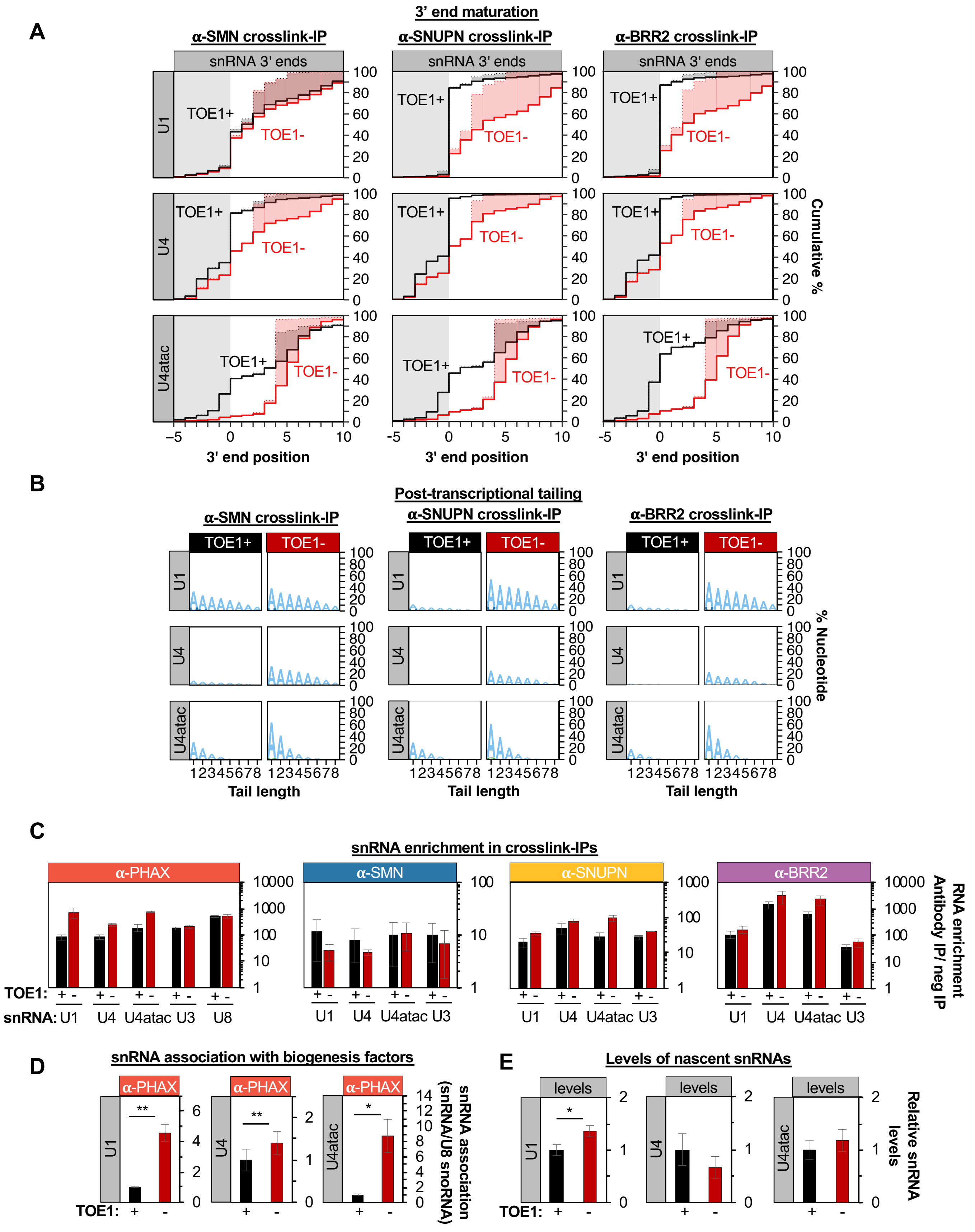

Supplemental Figure S3 related to Figure 3. TOE1 depletion causes accumulation of unprocessed adenylated snRNAs with PHAX. (A) Cumulative plots of 3’ end positions of U1, U4 and U4atac snRNAs associated with SMN, SNUPN and BRR2 after crosslinking/immunoprecipitation and 3’ end sequencing from cells with TOE1 present (black) or depleted (red). The average of three independent experiments is plotted. **(B)** Sequence logo plots representing the percent of snRNAs associated with SMN, SNUPN and BRR2 containing post-transcriptionally added tails, broken down by nucleotide composition, in the presence or absence of TOE1. The average of three independent experiments is plotted. **(C)** Enrichment of U1, U4, U4atac, U3 and U8 sn/snoRNAs in crosslinking and immunoprecipitation experiments using antibodies against PHAX, SMN, SNUPN, and BRR2 as measured by RT-qPCR and normalized to immunoprecipitation with no antibody, with the latter condition set to 1. Experiments were performed from cell lines with TOE1 present (black) or depleted (red). **(D)** Relative levels of U1, U4 and U4atac snRNAs associated with PHAX when TOE1 is present (black) or depleted (red) as measured by RT-qPCR assays normalized to the TOE1 non-target control U8 snoRNA, with averages of normalized U1, U4 and U4atac snRNA levels when TOE1 is present set to 1. **(E)** Relative levels of nascent U1, U4 and U4atac snRNAs in cells +/- TOE1, measured by RT-qPCR. Error bars: SEM from at least three independent experiments and p-values (Student’s two-tailed t-test) *:p<0.1; **:p <0.05; ***:p <0.01.

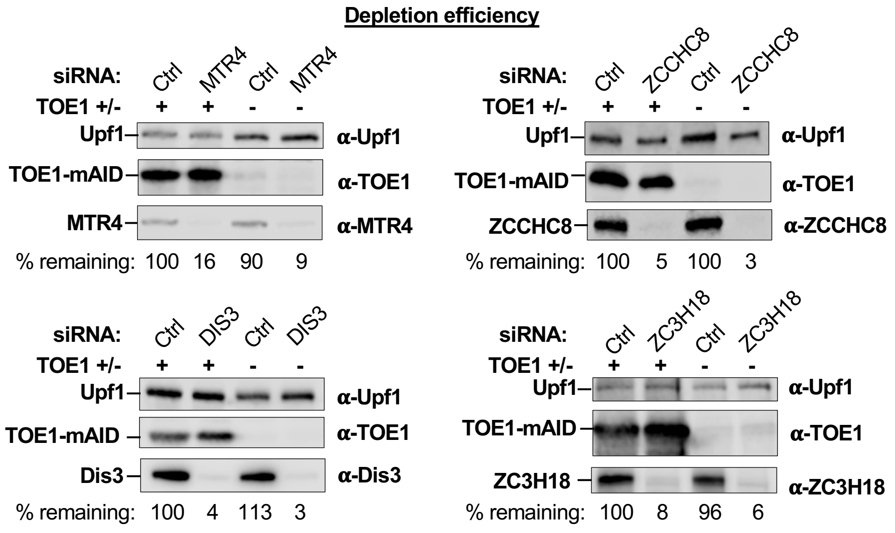

Supplemental Figure S4 related to Figure 4. snRNAs become targets of the nuclear exosome in the absence of TOE1. Representative Western blots showing co-depletions of MTR4, ZCCHC8, DIS3, and ZC3H18 with TOE1. Upf1 serves as an internal control for calculating percent remaining protein indicated below panels.

Supplemental Fig. S5

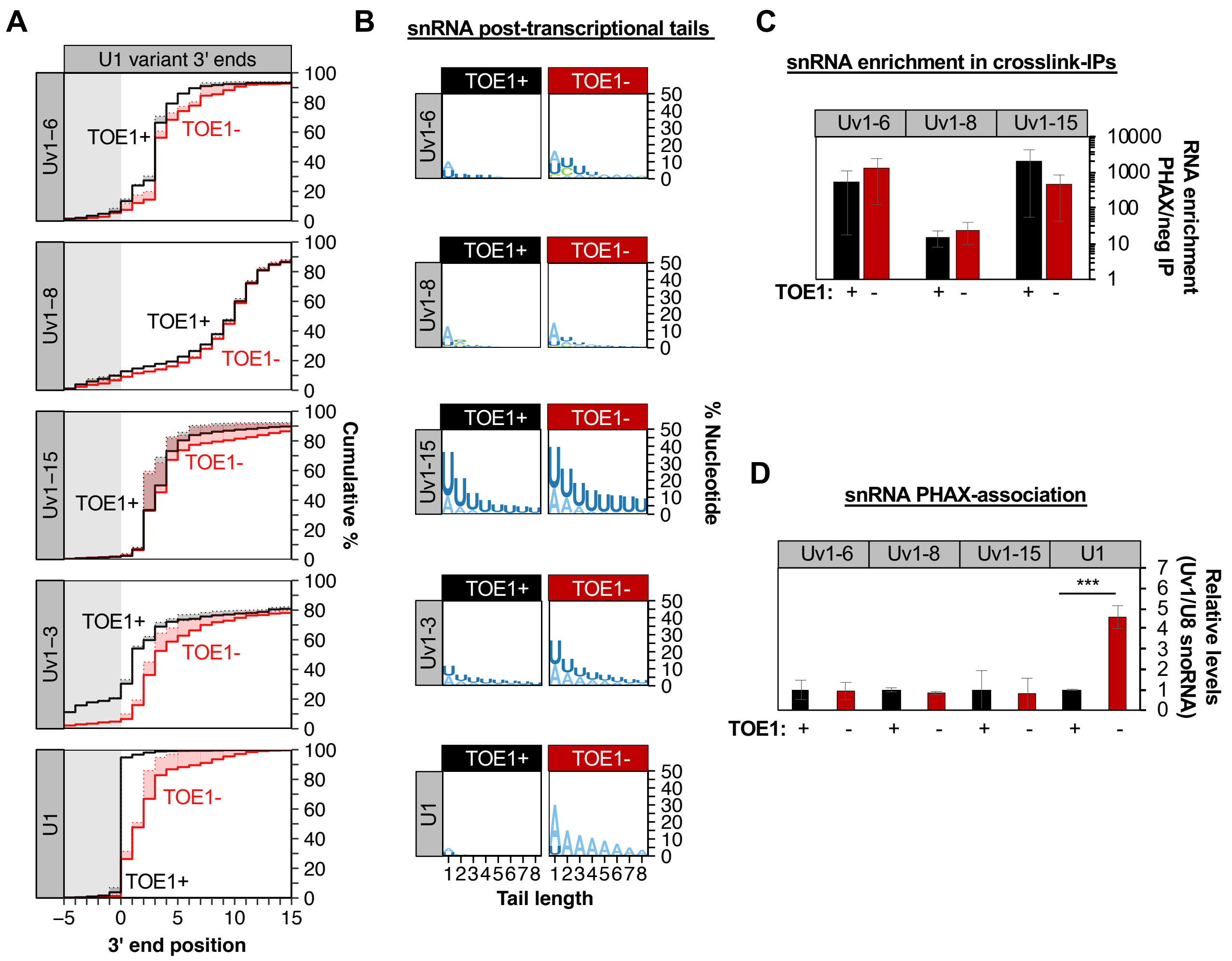

Supplemental Figure S5 related to Figure 5. TOE1 selectively processes regular U1 snRNA over U1 snRNA variants. (A) Cumulative plots of 3' ends of U1 variant snRNAs from RNA harvested at steady state when TOE1 is present (black) or depleted (red). The average of four independent experiments is plotted. **(B)** Sequence logo plots representing the percent U1 snRNA variants with post-transcriptionally added nucleotides when TOE1 is present or depleted, broken down by nucleotide composition. The average of four independent experiments is plotted. **(C)** Relative enrichment of snRNA variants with PHAX monitored by crosslinking/immunoprecipitation followed by RT-qPCR as normalized to immunoprecipitations with no antibody which was set to 1. **(D)** Relative levels of association of U1 variants with PHAX

when TOE1 is present (black) or depleted (red) as measured by RT-qPCR assays normalized to the TOE1 non-target control U8 snoRNA, with averages of normalized U1 variant snRNA levels when TOE1 is present set to 1. Error bars: SEM from three independent experiments and p-values (Student's two-tailed t-test) ***:$p < 0.01$.

Supplemental Fig. S6

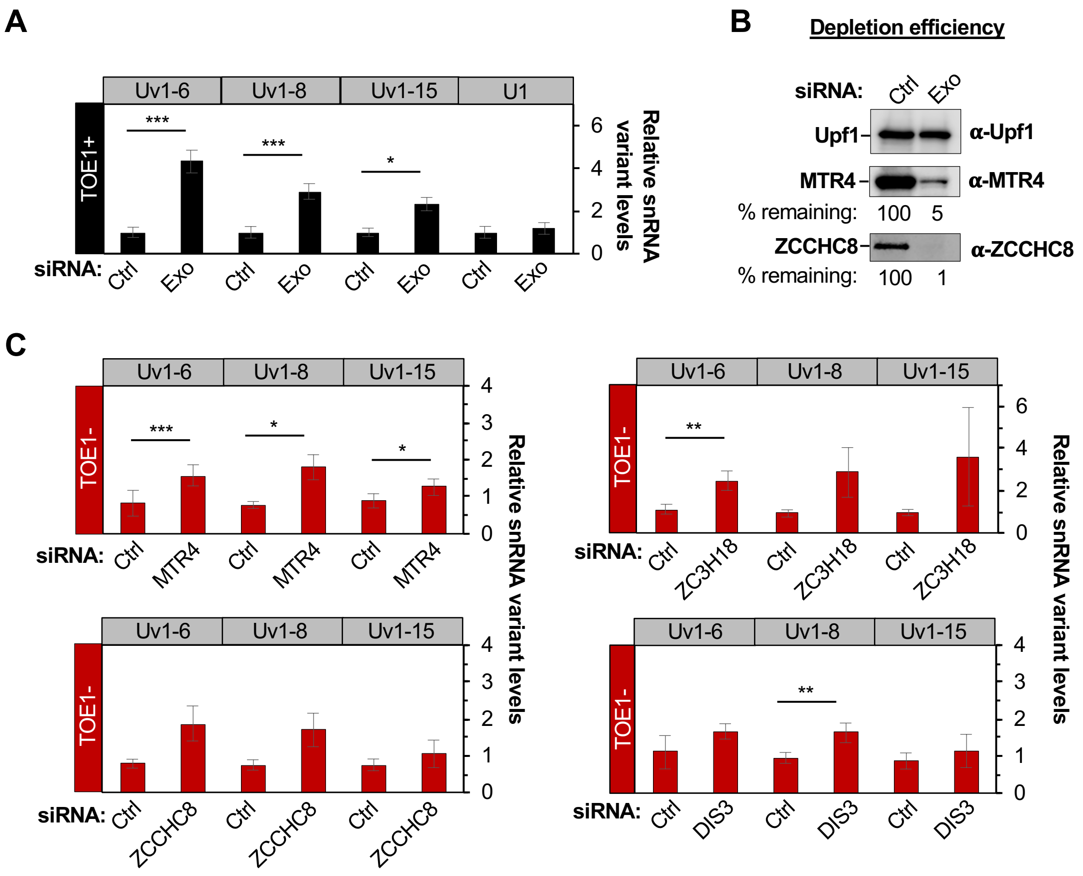

Supplemental Figure S6 related to Figure 6. U1 variant snRNAs are targets of the nuclear exosome. (A) Relative levels of variant U1 snRNAs upon siRNA-mediated co-depletion of MTR4 and ZCCHC8 as measured by RT-qPCR from total RNA and normalized to levels from control-treated (siCtrl) cells and to averages of 12S and 7SK RNA levels as internal controls. Error bars: SEM from four independent experiments and p-values (Student's two-tailed t-test) *:p<0.1; **:p <0.05; ***:p <0.01. **(B)** Representative western blots showing siRNA-medicated co-depletion of MTR4 and ZCCHC8. Upf1 is used an internal control for calculating percent remaining protein. **(C)** Relative levels of U1 variant snRNAs upon TOE1 depletion and siRNA-mediated depletion of Ctrl and MTR4, ZCCHC8, ZC3H18, or DIS3 as measured by RT-qPCR from total RNA and normalized to levels in the Ctrl siRNA/TOE1+ condition shown in Figure 6 and to the averages of mitochondrial 12S and 7SK RNA levels as internal controls. Error bars: SEM from at least

three independent experiments and p-values (Student's two-tailed t-test) *:$p<0.1$; **:$p<0.05$; ***:$p<0.01$.

Supplemental Table S1: RNA and DNA oligos used.

**RNA adapter**	**RNA sequence**
AG-10N	/5Phos/AGNNNNNNNNNNNAGAUCGGAAGAGCGUCGUG/3SpC3/
AG-11N	/5Phos/AGNNNNNNNNNNNNAGAUCGGAAGAGCGUCGUG/3SpC3/
**gene specific primers for 3' end library preparation**	
FU1Vs-01	CAGACGTGTGCTCTTCCGATCT ATCG ATGATCACGAAGGTGGTTTT
FU1Vs-02	CAGACGTGTGCTCTTCCGATCT GAACG ATGATCACGAAGGTGGTTTT
FU1Vs-03	CAGACGTGTGCTCTTCCGATCT GTTGTA ATGATCACGAAGGTGGTTTT
FU1Vs-04	CAGACGTGTGCTCTTCCGATCT CTACCAT ATGATCACGAAGGTGGTTTT
FU1Vs-05	CAGACGTGTGCTCTTCCGATCT TGGCTTCA ATGATCACGAAGGTGGTTTT
FU1Vs-06	CAGACGTGTGCTCTTCCGATCT ACTACGTGT ATGATCACGAAGGTGGTTTT
FU1-Vs-07	CAGACGTGTGCTCTTCCGATCT TCGTACACAG ATGATCACGAAGGTGGTTTT
FU1-Vs-08	CAGACGTGTGCTCTTCCGATCT CACAGTATGAC ATGATCACGAAGGTGGTTTT
FU1-Vs-09	CAGACGTGTGCTCTTCCGATCT ATGCTGACAAGT ATGATCACGAAGGTGGTTTT
FU1-Vs-10	CAGACGTGTGCTCTTCCGATCT GTCAAGTGCTAGC ATGATCACGAAGGTGGTTTT
FU1-Vs-11	CAGACGTGTGCTCTTCCGATCT CAATGTACGAACGT ATGATCACGAAGGTGGTTTT
FU1-Vs-12	CAGACGTGTGCTCTTCCGATCT TAGC ATGATCACGAAGGTGGTTTT
FU1-Vs-13	CAGACGTGTGCTCTTCCGATCT CTTGC ATGATCACGAAGGTGGTTTT
FU1-Vs-14	CAGACGTGTGCTCTTCCGATCT CAACAT ATGATCACGAAGGTGGTTTT
FU1-Vs-15	CAGACGTGTGCTCTTCCGATCT GATGGTA ATGATCACGAAGGTGGTTTT
FU1-Vs-16	CAGACGTGTGCTCTTCCGATCT ACCGAAGT ATGATCACGAAGGTGGTTTT
FU1-Vs-17	CAGACGTGTGCTCTTCCGATCT TGATGCACA ATGATCACGAAGGTGGTTTT
FU1-Vs-18	CAGACGTGTGCTCTTCCGATCT AGCATGTGTC ATGATCACGAAGGTGGTTTT
FU1-Vs-19	CAGACGTGTGCTCTTCCGATCT GTGTCATACTG ATGATCACGAAGGTGGTTTT
FU1-Vs-20	CAGACGTGTGCTCTTCCGATCT TACGACTGTTCA ATGATCACGAAGGTGGTTTT
FU1-Vs-21	CAGACGTGTGCTCTTCCGATCT CAGTTCACGATCG ATGATCACGAAGGTGGTTTT
FU1-Vs-22	CAGACGTGTGCTCTTCCGATCT GTTACATGCTTGCA ATGATCACGAAGGTGGTTTT
FU1-Vs-23	CAGACGTGTGCTCTTCCGATCT TTCCA ATGATCACGAAGGTGGTTTT
FU1-Vs-24	CAGACGTGTGCTCTTCCGATCT AAGGTC ATGATCACGAAGGTGGTTTT
FU1-Vs-25	CAGACGTGTGCTCTTCCGATCT CCTTGAG ATGATCACGAAGGTGGTTTT
FU1-Vs-26	CAGACGTGTGCTCTTCCGATCT GGAACTCG ATGATCACGAAGGTGGTTTT
FU1-Vs-27	CAGACGTGTGCTCTTCCGATCT ACCTG ATGATCACGAAGGTGGTTTT
FU1-Vs-28	CAGACGTGTGCTCTTCCGATCT TGGACC ATGATCACGAAGGTGGTTTT
FU1-Vs-29	CAGACGTGTGCTCTTCCGATCT CTTGAGC ATGATCACGAAGGTGGTTTT
FU1-Vs-30	CAGACGTGTGCTCTTCCGATCT GAATCTAC ATGATCACGAAGGTGGTTTT
FU1-Vs-31	CAGACGTGTGCTCTTCCGATCT TTGGC ATGATCACGAAGGTGGTTTT
FU1-Vs-32	CAGACGTGTGCTCTTCCGATCT AACTGGC ATGATCACGAAGGTGGTTTT
FU1-Vs-33	CAGACGTGTGCTCTTCCGATCT CAACCGAT ATGATCACGAAGGTGGTTTT
FU1-Vs-34	CAGACGTGTGCTCTTCCGATCT GTTGGAATC ATGATCACGAAGGTGGTTTT
FU1-Vs-35	CAGACGTGTGCTCTTCCGATCT CCGGA ATGATCACGAAGGTGGTTTT
FU1-Vs-36	CAGACGTGTGCTCTTCCGATCT TCTCTA ATGATCACGAAGGTGGTTTT
FU4-01	CAGACGTGTGCTCTTCCGATCT CGTA GCAGTATCGTAGCCAATGAGG
FU4-02	CAGACGTGTGCTCTTCCGATCT CGTTC GCAGTATCGTAGCCAATGAGG
FU4-03	CAGACGTGTGCTCTTCCGATCT TACAAC GCAGTATCGTAGCCAATGAGG
FU4-04	CAGACGTGTGCTCTTCCGATCT ATGGTAG GCAGTATCGTAGCCAATGAGG
FU4-05	CAGACGTGTGCTCTTCCGATCT TGATGGCA GCAGTATCGTAGCCAATGAGG
FU4-06	CAGACGTGTGCTCTTCCGATCT ACACGTTGT GCAGTATCGTAGCCAATGAGG
FU4atac-01	CAGACGTGTGCTCTTCCGATCT ACCAC CCATCTTTTTCTTGAGGTTGCAC
FU4atac-02	CAGACGTGTGCTCTTCCGATCT GCACTA CCATCTTTTTCTTGAGGTTGCAC
FU4atac-03	CAGACGTGTGCTCTTCCGATCT GTGACTA CCATCTTTTTCTTGAGGTTGCAC
FU4atac-04	CAGACGTGTGCTCTTCCGATCT TTCTCTA CCATCTTTTTCTTGAGGTTGCAC
FU4atac-05	CAGACGTGTGCTCTTCCGATCT CACGT CCATCTTTTTCTTGAGGTTGCAC
FU4atac-06	CAGACGTGTGCTCTTCCGATCT TTCACTAT CCATCTTTTTCTTGAGGTTGCAC
FU2-01	CAGACGTGTGCTCTTCCGATCT TACG GGAGATGGAATAGGAGCTTGC
FU2-02	CAGACGTGTGCTCTTCCGATCT CTGCT GGAGATGGAATAGGAGCTTGC
FU2-03	CAGACGTGTGCTCTTCCGATCT CAATCA GGAGATGGAATAGGAGCTTGC
FU2-04	CAGACGTGTGCTCTTCCGATCT GTAGATG GGAGATGGAATAGGAGCTTGC
FU2-05	CAGACGTGTGCTCTTCCGATCT AGCCATAG GGAGATGGAATAGGAGCTTGC
FU2-06	CAGACGTGTGCTCTTCCGATCT TGTACAGCA GGAGATGGAATAGGAGCTTGC
FU2-07	CAGACGTGTGCTCTTCCGATCT TCGG GGAGATGGAATAGGAGCTTGC
FU2-08	CAGACGTGTGCTCTTCCGATCT CGACA GGAGATGGAATAGGAGCTTGC
FU2-09	CAGACGTGTGCTCTTCCGATCT CATTGA GGAGATGGAATAGGAGCTTGC
FU2-10	CAGACGTGTGCTCTTCCGATCT GCGAAGT GGAGATGGAATAGGAGCTTGC
FU2-11	CAGACGTGTGCTCTTCCGATCT ACTAGTCA GGAGATGGAATAGGAGCTTGC
FU2-12	CAGACGTGTGCTCTTCCGATCT TGAAGCATA GGAGATGGAATAGGAGCTTGC
FU5-04	CAGACGTGTGCTCTTCCGATCT GATGGTA ATACTCTGGTTTCTCTTCAGATCG
FU5-05	CAGACGTGTGCTCTTCCGATCT ACCGAAGT ATACTCTGGTTTCTCTTCAGATCG
FU5-06	CAGACGTGTGCTCTTCCGATCT TGATGCACA ATACTCTGGTTTCTCTTCAGATCG
FU5-07	CAGACGTGTGCTCTTCCGATCT GACATTCGTA ATACTCTGGTTTCTCTTCAGATCG
FU5-08	CAGACGTGTGCTCTTCCGATCT ATGGACTAGTA ATACTCTGGTTTCTCTTCAGATCG
FU5-09	CAGACGTGTGCTCTTCCGATCT ATCATG ATACTCTGGTTTCTCTTCAGATCG
FU5-10	CAGACGTGTGCTCTTCCGATCT TAGCATG ATACTCTGGTTTCTCTTCAGATCG
FU11-01	CAGACGTGTGCTCTTCCGATCT GATC CGACATCAAGAGATTTCGGAAGC
FU11-02	CAGACGTGTGCTCTTCCGATCT TGAAG CGACATCAAGAGATTTCGGAAGC
FU11-03	CAGACGTGTGCTCTTCCGATCT CTATAC CGACATCAAGAGATTTCGGAAGC
FU11-04	CAGACGTGTGCTCTTCCGATCT ACGACTG CGACATCAAGAGATTTCGGAAGC
FU11-05	CAGACGTGTGCTCTTCCGATCT TCATGACA CGACATCAAGAGATTTCGGAAGC
FU11-06	CAGACGTGTGCTCTTCCGATCT AGTCAGTTG CGACATCAAGAGATTTCGGAAGC
FU12-01	CAGACGTGTGCTCTTCCGATCT CTAG CGAATCCTCACTGCTAATGTGAG
FU12-02	CAGACGTGTGCTCTTCCGATCT ACTTC CGAATCCTCACTGCTAATGTGAG
FU12-03	CAGACGTGTGCTCTTCCGATCT GATATG CGAATCCTCACTGCTAATGTGAG
FU12-04	CAGACGTGTGCTCTTCCGATCT TGCTGAC CGAATCCTCACTGCTAATGTGAG
FU12-05	CAGACGTGTGCTCTTCCGATCT AGTACTGT CGAATCCTCACTGCTAATGTGAG
FU12-06	CAGACGTGTGCTCTTCCGATCT TCAGTCAAC CGAATCCTCACTGCTAATGTGAG
FU3-01	CAGACGTGTGCTCTTCCGATCT TACA CTCTGAACGTGTAGAGCAC
FU3-02	CAGACGTGTGCTCTTCCGATCT ATGTA CTCTGAACGTGTAGAGCAC
FU3-03	CAGACGTGTGCTCTTCCGATCT GCACGA CTCTGAACGTGTAGAGCAC

Supplemental Table S1: RNA and DNA oligos used.

FU3-04	CAGACGTGTGCTCTTCCGATCT AGTGCTA CTCTGAACGTGTAGAGCAC
FU3-05	CAGACGTGTGCTCTTCCGATCT CAACTCGA CTCTGAACGTGTAGAGCAC
FU3-06	CAGACGTGTGCTCTTCCGATCT TCTTGGATA CTCTGAACGTGTAGAGCAC
FU8-01	CAGACGTGTGCTCTTCCGATCT AATC GGATAATCCTTACCTGTTCCTCC
FU8-02	CAGACGTGTGCTCTTCCGATCT TTCGA GGATAATCCTTACCTGTTCCTCC
FU8-03	CAGACGTGTGCTCTTCCGATCT CCAAAA GGATAATCCTTACCTGTTCCTCC
FU8-04	CAGACGTGTGCTCTTCCGATCT GGTTTTG GGATAATCCTTACCTGTTCCTCC
FU1V68-01	CAGACGTGTGCTCTTCCGATCT ATGC TTCCCCAAATGTGGGAA
FU1V68-02	CAGACGTGTGCTCTTCCGATCT GACGA TTCCCCAAATGTGGGAA
FU1V68-03	CAGACGTGTGCTCTTCCGATCT GTTAGT TTCCCCAAATGTGGGAA
FU1V68-04	CAGACGTGTGCTCTTCCGATCT CATCTAC TTCCCCAAATGTGGGAA
FU1V68-05	CAGACGTGTGCTCTTCCGATCT TCGGTATC TTCCCCAAATGTGGGAA
FU1V68-06	CAGACGTGTGCTCTTCCGATCT ACATGTCGT TTCCCCAAATGTGGGAA
FUv1-6-01	CAGACGTGTGCTCTTCCGATCT AGGA TGGCAGGAGAGATACCCTGG
FUv1-6-02	CAGACGTGTGCTCTTCCGATCT TGGT TGGCAGGAGAGATACCCTGG
FUv1-6-03	CAGACGTGTGCTCTTCCGATCT ACACA TGGCAGGAGAGATACCCTGG
FUv1-6-04	CAGACGTGTGCTCTTCCGATCT GACGA TGGCAGGAGAGATACCCTGG
FUv1-6-05	CAGACGTGTGCTCTTCCGATCT CCAATT TGGCAGGAGAGATACCCTGG
FUv1-6-06	CAGACGTGTGCTCTTCCGATCT GGAATA TGGCAGGAGAGATACCCTGG
FUv1-3-01	CAGACGTGTGCTCTTCCGATCT TGTG ATACTTATGTTTATCTGGCAGAAGAA
FUv1-3-02	CAGACGTGTGCTCTTCCGATCT CACG ATACTTATGTTTATCTGGCAGAAGAA
FUv1-3-03	CAGACGTGTGCTCTTCCGATCT GCCAT ATACTTATGTTTATCTGGCAGAAGAA
FUv1-3-04	CAGACGTGTGCTCTTCCGATCT ATACG ATACTTATGTTTATCTGGCAGAAGAA
FUv1-3-05	CAGACGTGTGCTCTTCCGATCT TTACCG ATACTTATGTTTATCTGGCAGAAGAA
FUv1-3-06	CAGACGTGTGCTCTTCCGATCT AATGGC ATACTTATGTTTATCTGGCAGAAGAA
FUv1-15-01	CAGACGTGTGCTCTTCCGATCT AAGG ATCCGTTATGTTCCGGATGTA
FUv1-15-02	CAGACGTGTGCTCTTCCGATCT TTCC ATCCGTTATGTTCCGGATGTA
FUv1-15-03	CAGACGTGTGCTCTTCCGATCT GGTTA ATCCGTTATGTTCCGGATGTA
FUv1-15-04	CAGACGTGTGCTCTTCCGATCT CCAAT ATCCGTTATGTTCCGGATGTA
FUv1-15-05	CAGACGTGTGCTCTTCCGATCT GAGTCT ATCCGTTATGTTCCGGATGTA
FUv1-15-06	CAGACGTGTGCTCTTCCGATCT ACAGTG ATCCGTTATGTTCCGGATGTA
FUv1-8-03	CAGACGTGTGCTCTTCCGATCT CAGTTG CCTAGCAGAAGAAAATCGTGTTTACG
FUv1-8-04	CAGACGTGTGCTCTTCCGATCT TCCAAAT CCTAGCAGAAGAAAATCGTGTTTACG
FUv1-8-05	CAGACGTGTGCTCTTCCGATCT ATTCCAAT CCTAGCAGAAGAAAATCGTGTTTACG
FUv1-8-06	CAGACGTGTGCTCTTCCGATCT GATTGGCCT CCTAGCAGAAGAAAATCGTGTTTACG
**library prep and sequencing**	
AR-17	ACACGACGCTCTTCCGA
PCR_F_D501	AATGATACGGCGACCACCGAGATCTACACTATAGCCTACACTCTTTCCCTACACGACGCTCTT
PCR_F_D502	AATGATACGGCGACCACCGAGATCTACACATAGAGGCACACTCTTTCCCTACACGACGCTCTT
PCR_F_D503	AATGATACGGCGACCACCGAGATCTACACCCTATCCTACACTCTTTCCCTACACGACGCTCTT
PCR_F_D701	CAAGCAGAAGACGGCATACGAGATCGAGTAATGTGACTGGAGTTCAGACGTGTGCTCTTCCGA
PCR_F_D702	CAAGCAGAAGACGGCATACGAGATTCTCCGGAGTGACTGGAGTTCAGACGTGTGCTCTTCCGA
PCR_F_D703	CAAGCAGAAGACGGCATACGAGATAATGAGCGGTGACTGGAGTTCAGACGTGTGCTCTTCCGA
PCR_F_D704	CAAGCAGAAGACGGCATACGAGATGGAATCTCGTGACTGGAGTTCAGACGTGTGCTCTTCCGA
PCR_F_D705	CAAGCAGAAGACGGCATACGAGATTTCTGAATGTGACTGGAGTTCAGACGTGTGCTCTTCCGA
PCR_F_D706	CAAGCAGAAGACGGCATACGAGATACGAATTCGTGACTGGAGTTCAGACGTGTGCTCTTCCGA
**gRNAs**	
CAF1Z_gRNA1	GACACCACCTGGGGGTTAGG
CAF1Z_gRNA2	TGCTTTTGCTGACACCACCT
CAF1Z_gRNA3	GCTGTGGACACGGTGAGAGT
**primers - endogeous knockout**	
caf1z_seq_F1	ACGTGGGTTACAAAGGCTT
caf1z_seq_F2	GCTTGTGGGAAAAGCTACCA
caf1z_seq_R1	TTCAGCAAACTCTTCCTGTCC
caf1z_seq_R2	ACCTACCCACAGGAGCTGAG
**siRNAs**	
siControl (siLuciferase)	CGUACGCGGAAUACUUCGAUU
siMTR4	CAAUUAAGGCUCUGAGUAAUU
siZCCHC8	GGAAUGUACCUCAGGAUAAUU
siZC3H18	GGAAUGAAUUGUAGGUUUAUU
siDIS3	AGGUAGAGUUGUAGGAAUAUU
**qPCR primers**	
U1-forward	GCACTCCGGATGTGCTGACCC
U1-reverse	CAGGGGAAAGCGCGAACGCAG
U4-forward	GCGCGATTATTGCTAATTGAAA
U4-reverse	AAAAATTGCCAATGCCGACTA
U4atac-forward	GCGCATAGTGAGGGCAGTACT
U4atac-reverse	GCACCAAAATAAAGCAAAAGCTCTA
7sk-forward	GAGGGCGATCTGGCTGCGACAT
7sk-reverse	ACATGGAGCGGTGAGGGAGGAA
12s-forward	ATGCAGCTCAAAACGCTTAGC
12s-reverse	GCTGGCACGAAATTGACCAA
Uv1-6-forward	TGGCAGGAGAGATACCCTGG
Uv1-6-reverse	CGCGAATGCAGTCTACCACTG
Uv1-8-forward	GAAGCTAATTCGTGCAACTTCCC
Uv1-8-reverse	CGGGGGAAAAGAGCGAACGCAG
Uv1-15-forward	GTTATGTTCCGGGTGTACTGACCCCTGCC
Uv1-15-reverse	CAGTCGAGTTCCTCACATTG
U3-forward	AGAGGTAGCGTTTTCTCCTGAGCG
U3-reverse	ACCACTCAGACCGCGTTCTC
U8-forward	CGTCAGGTGGGATAATCCTT
U8-reverse	GGGTGTTGCAAGTCCTGATT
